# The calcium channel Orai1 is required for osteoblast development: Studies in a chimeric mouse with variable *in vivo *Runx-cre deletion of Orai-1

**DOI:** 10.1371/journal.pone.0264596

**Published:** 2023-05-11

**Authors:** Lisa J. Robinson, Jonathan Soboloff, Irina L. Tourkova, Quitterie C. Larrouture, Kelechi M. Onwuka, Dionysios J. Papachristou, Scott Gross, Robert Hooper, Elsie Samakai, Paul F. Worley, Peng Liu, Jan Tuckermann, Michelle R. Witt, Harry C. Blair

**Affiliations:** 1 Departments of Pathology, Anatomy and Laboratory Medicine, and of Microbiology, Immunology & Cell Biology, West Virginia University School of Medicine, Morgantown, WV, United States of America; 2 Fels Cancer Institute for Personalized Medicine, Department of Cancer and Cellular Biology, Lewis Katz School of Medicine at Temple University, Philadelphia, PA, United States of America; 3 Departments of Pathology and of Cell Biology, The Pittsburgh VA Medical Center and the University of Pittsburgh, Pittsburgh, PA, United States of America; 4 Laboratory of Bone and Soft Tissue Studies, Department of Anatomy-Histology-Embryology, University Patras Medical School, Patras, Greece; 5 Solomon H. Snyder Department of Neuroscience, Johns Hopkins University School of Medicine, Baltimore, MD, United States of America; 6 Institute of Comparative Molecular Endocrinology, Helmholtzstraße, Ulm, Germany; Indiana University School of Medicine, UNITED STATES

## Abstract

The calcium-selective ion channel Orai1 has a complex role in bone homeostasis, with defects in both bone production and resorption detected in Orai1 germline knock-out mice. To determine whether Orai1 has a direct, cell-intrinsic role in osteoblast differentiation and function, we bred Orai1 flox/flox (Orai1^fl/fl^) mice with Runx2-cre mice to eliminate its expression in osteoprogenitor cells. Interestingly, Orai1 was expressed in a mosaic pattern in Orai1^fl/fl^-Runx2-cre bone. Specifically, antibody labeling for Orai1 in vertebral sections was uniform in wild type animals, but patchy regions in Orai1^fl/fl^-Runx2-cre bone revealed Orai1 loss while in other areas expression persisted. Nevertheless, by micro-CT, bones from Orai1^fl/fl^-Runx2-cre mice showed reduced bone mass overall, with impaired bone formation identified by dynamic histomorphometry. Cortical surfaces of Orai1^fl/fl^-Runx2-cre vertebrae however exhibited patchy defects. In cell culture, Orai1-negative osteoblasts showed profound reductions in store-operated Ca^2+^ entry, exhibited greatly decreased alkaline phosphatase activity, and had markedly impaired substrate mineralization. We conclude that defective bone formation observed in the absence of Orai1 reflects an intrinsic role for Orai1 in differentiating osteoblasts.

## Introduction

Orai1 is a calcium-selective ion channel found in the plasma membrane, activated by STIM1, a calcium sensor located on the endoplasmic reticulum [1–3]. Although expressed in all cells, the extent to which different cell types require Orai1 is variable. For example, T cell activation requires Orai1; its loss causes severe combined immunodeficiency [4, 5]. Loss of Orai1 also causes a wide range of changes in cardiovascular function [6], neuronal function [7, 8], tooth formation [9, 10] and others [11]. We [12–16] and others [17, 18], showed that Orai1 contributes to bone differentiation and maintenance in regular knockout mice and *in vitro* cell lines.

Investigation of mice with global deletion of Orai1 revealed loss of multinucleated osteoclasts, that, intriguingly, did not lead to osteopetrosis: Instead, micro-computed tomography showed reduced cortical ossification and thinned trabeculae in Orai1^-/-^ animals compared with controls [12]. We attributed the unexpected finding of reduced osteoclast formation without increased bone density to a probable defect in bone formation by osteoblasts. However, considering the near-lethal phenotype of Orai1^-/-^ mice [11] and the interdependence of osteoclast and osteoblast differentiation, these investigations were insufficient to draw definitive conclusions regarding the role of Orai1 within bone-forming osteoblasts specifically.

Here, we assessed the role of Orai1 on osteoblast differentiation *in vivo* using a conditional knockout mouse. Interestingly, we found that Orai1 deletion *in vivo* was highly regional, creating a mosaic effect within bone. Irrespective, loss of Orai1 eliminated store-operated Ca^2+^ entry in osteoblasts and precursors resulted in a severe defect in osteoblast differentiation and function. This shows that Orai1 is required for normal osteoblast function.

## Materials and methods

### Animals and genotyping

Orai1^fl/fl^ mice were generated by flanking Orai1 exons 2/3 with loxP as previously described [16, 19]. Mice were backcrossed to C57Bl/6 for a minimum of 10 generations and then crossed with Runx2-cre mice (Tg(Runx2-icre)1Jtuc) [20] for conditional deletion in the osteoblast lineage. Orai1^fl/fl^ mice were identified by PCR of genomic DNA from tail snips using the following primers: flox-F: acc cat gtg gtg gaa aga aa and flox-R: tgc agg cac taa aga cga tg; these generate a 746 bp product in wild-type mice and a 505 bp product in Orai1^fl/fl^ mice [16]. To confirm excision of Orai1 from bone cells, PCR of genomic DNA was performed using the flox-F primer paired with excision-R: cag aaa gaa cta cac aga gaa atc, as described [16]; excision results in a 520 bp product, while none is produced when the gene is intact. Mice were sacrificed at 16 weeks unless otherwise noted. For dynamic histomorphometry, animals were injected with xylenol orange (80 μg/g mouse weight) five days prior to sacrifice, then with calcein (20 μg/g mouse weight) 2 days later. Mouse long bones were dissected, cleaned of soft tissue, and processed for isolation of mesenchymal stem cells and osteoblasts. The vertebral column was removed for histologic and microCT analysis. Work was approved by the Temple University IACUC. All animals for study shown contained floxed Orai1 (Orai1^fl/fl^); those with the cre recombinase are shown as Orai1^fl/fl^ RunX2-cre conditional KO (cKO).

### Cell isolation and differentiation

Unless stated, media and chemicals were from Thermo-Fisher. Mesenchymal stem cells (MSC) were isolated from long bones as described [21]. Briefly, marrow was flushed with Minimum Essential Medium-alpha (alphaMEM) containing 10% fetal bovine serum (Sigma-Aldrich, St. Louis, MO), penicillin and streptomycin. After plating cells 16 hours to allow adhesion of fibroblasts, non-adherent cells were collected and re-plated in RPMI medium supplemented with containing 10% fetal bovine serum, penicillin, and streptomycin for 3 to 4 days. Non-adherent cells were discarded, and the adherent cells grown in selective medium (Mesencult, StemCell Technologies, Cambridge, MA), at 2 x 10^6^ cells/ cm^2^, for proliferation of MSC. Osteoblasts were isolated from long bones as described [22]: briefly, after removal of marrow, the long bones, cut into 1–2 mm fragments, were incubated in 2 mg/ml collagenase type II (260 U/mg, Worthington, Lakewood, NJ) in Dulbecco’s Modified Eagle’s Medium (DMEM) for 2 hours at 37°C. The bone fragments were then rinsed and transferred to culture dishes with Osteoblast Growth Medium (DMEM with 1 g/l glucose, 10% heat inactivated fetal bovine serum, 30 μg/ml ascorbate, penicillin, streptomycin, and amphotericin B. For differentiation to osteoblasts with mineralized matrix formation, MSC or osteoblasts at confluence were transferred to Osteoblast Mineralization Medium (Osteoblast Growth medium with 30 μg/ml ascorbate and 10 mM 2-glycerol phosphate). During culture, media were replaced every 2–3 days.

### Histomorphometry and histology

Static histomorphometry was performed as described [16]. Briefly, lumbar vertebrae were fixed overnight in 3.7% formalin, then transferred to 70% ethanol for micro-CT analysis using Bruker Skyscan 1272 with a bone density cutoff of 150 mg/cm^2^, at 5 μm resolution. Scans were analyzed using Bruker CTan software for trabecular parameters; three-dimensional images were produced with Bruker CTvox software. For dynamic histomorphometry, vertebral sections were taken from animals labeled with xylenol orange and calcein two days apart. Lumbar vertebrae 1–3 were used for dynamic histomorphometry and histologic studies, ribs 5–6, and vertebrae 4–6 were for micro-computed tomography. Bone samples for fluorescent microscopy were cut without decalcification. For hematoxylin and eosin staining, bone was fixed, dehydrated, paraffin embedded, and cut as 10 μm thick sections using a rotary microtome [23]. Cortical thickness was measured orthogonal to the vertical axis in microns at 300 μm intervals; dynamic histomorphometry was as described using calcein and xylenol orange labels [23]. Measurements were labeling per surface area and inter-label difference for bone formation rate. Labeling was analyzed by observers blinded to genotype.

### Degraded bone and osteoblast active and inactive surface

Bone surface was evaluated using conditional KO and control sections for areas degraded by scalloped surface, using the same sections produced for xylenol orange and calcein labeling. Osteoblast surface area (bone formation surface) (Ob.S/BS) was estimated as cuboidal bone lining cell area, on phase contrast photographs of sections of trabecular bone, as reported [16]. This measurement was blinded relative to measurement of fluorescent labels.

### Alkaline phosphatase, mineralization, and adipocyte assays, and PCR for characteristic proteins in vitro

Mineral was labeled with silver nitrate (von Kossa stain): cultures were rinsed with water and fixed with 3.7% formalin for 2 minutes, then incubated with 2% AgNO_3_ under UV light for 10 minutes, and rinsed again with water. Alkaline phosphatase activity was determined in cell cultures using 0.01% naphthol phosphate substrate in citrate-buffered saline at pH 8 plus 0.25 mg/ml of fast blue to precipitate an insoluble blue adduct [24]. Parallel cultures were assessed for adipocyte formation by incubating fixed cells in 0.3% Oil red O in 60% isopropanol for 1 hour and rinsed with water. Proteins characteristic of osteoblasts were assayed by real time PCR as described [16]. In brief, RNA, by oligo dT affinity, was used for cDNA synthesis using random hexamers and MMLV reverse transcriptase. Quantitative PCR used an MX3000P instrument (Stratagene) with SYBR green to monitor DNA synthesis. Duplicate reactions were run in 25 μl volumes containing 12.5 μl of pre-mixed dye, NTPs, buffer, and polymerase, 250 nM primers and 1 μl of first strand cDNA. After 10 minutes at 95°C, the mixture was amplified in cycles of 30 seconds at 95°C, 30 seconds at 59°C, and 1 minute at 72°C. Product abundance relative to controls was calculated assuming linearity to log(initial copies). Primers were GAPDH: F—5'-GTTGTCTCCTGCGACTTCA -3' R—5'-GGTGGTCCAGGGTTTCTTA-3'.RunX2: F—5'-ATGATGACACTGCCACCTCTGAC-3' R—5'-ACTGCCTGGGGTCTGAAAAAGG-3'. Orai1: F—5'- TACTTAAGCCGCGCCAAGCT-3' R—5'GCAGGTGCTGATCATGAGGGC-3'. Alkaline phosphatase: F—5'-ATCGGAACAACCTGACTGACCCTT-3' R—5'-ACCCTCATGATGTCCGTGGTCAAT-3'. Col I a1: F—5'-TTCTCCTGGCAAAGACGGACTCAA-3' R—5'-AGGAAGCTGAAGTCATAACCGCCA-3'. Osteocalcin: F—5`-ACCATCTTTCTGCTCACTCTGCTG-3`R—5`-TATTGCCCTCCTGCTTGGACATGA-3`. Osterix (SP7): F—5'-GATGGCGTCCTCTCTGCTT-3', R—5'-CGTATGGCTTCTTTGTGCCT 3'. ATF4 (CREB2): F—5'-CCTGAACAGCGAAGTGTTGG-3', R—5'-TGGAGAACCCATGAGGTTTCAA-3'.

### Measurement of store operated calcium entry

Osteoblasts on glass coverslips were loaded with 2 μM Fura-2 AM-ester (Invitrogen) for 30 minutes at 25°C in 107 mM NaCl, 7.2 mM KCl, 1.2 mM MgCl_2_, 11.5 mM glucose, 20 mM HEPES, 1 mM CaCl_2_, at pH 7.2. Washed cells were allowed to de-esterify dye for 30 minutes at 25°C as described [16]. Ca^2+^ measurements used a Leica DMI 6000B fluorescence microscope controlled by Slidebook Software (Intelligent Imaging; Denver, CO). Intracellular Ca^2+^ is shown as 340/380 nm ratios from single cells. Data used 15–20 cells per mouse and three or more experiments.

#### Orai1 labeling

Rabbit polyclonal-anti Orai1 was used for tissue labeling [25]; rabbit anti-Orai1 (extracellular) antibody was from Alomone Labs (ACC-062, Jerusalem, Israel). Triplicate sections from three wild type and three knockout animals (to allow no-antibody controls) of wild Orai1^fl/fl^ and Orai1^fl/fl^ with RunX2-cre (cKO) were de-paraffinized by 67°C heating for two days followed by a xylene rinse 12 hours. Labeling was done twice with different sections. In each case, sections were hydrated with 70% ethanol followed by PBS with 1% bovine serum albumin (BSA) with 2 mM EDTA (blocking solution) overnight at room temperature, followed by antibody at 1:100 in PBS with BSA and EDTA (or no antibody controls) overnight at room temperature, and then secondary labeling with anti-rabbit Alexafluor 488 (green) for four hours at room temperature, followed by rinsing and fixation with 3.7% formalin in PBS. When indicated, post staining with phalloidin-rhodamine (1:100) was used to label actin. For Western blots, cell lysates were made using 0.3% SDS, 50 mM tris, pH 7, with proteinase, and phosphatase inhibitors. Proteins were separated on 4–12% gradient gel and transferred to polyvinylidene difluoride (PVDF) membranes. The primary antibody was the Alomone antibody used for fluorescence (1:200); secondary antibody was horse radish peroxidase conjugated anti-rabbit (1:40,000, Jackson ImmunoResearch, West Grove, PA). Proteins on blots were detected by enhanced chemiluminescence detection (ECL plus, Amersham, Piscataway, NJ, USA). Mouse beta-actin antibody (1:1,000, Sigma) with secondary horse radish peroxidase conjugated anti-mouse (1:40,000) were controls.

#### Statistics

Results are mean ± SD, for three or more measurements or as stated. Comparisons of differences used Student’s unpaired t-test. Significance indicates p 0.05 or less.

#### Supporting information

Genotypes, ages, and labeling of animals, micro-computed tomography, data for graphs, and original un-cropped and unadjusted images of blots are shown in Supporting Information appended to the paper (S1 Data).

## Results

### Phenotype of wild type and Orai1^fl/fl^-Runx2-cre animals

**Fig 1:**
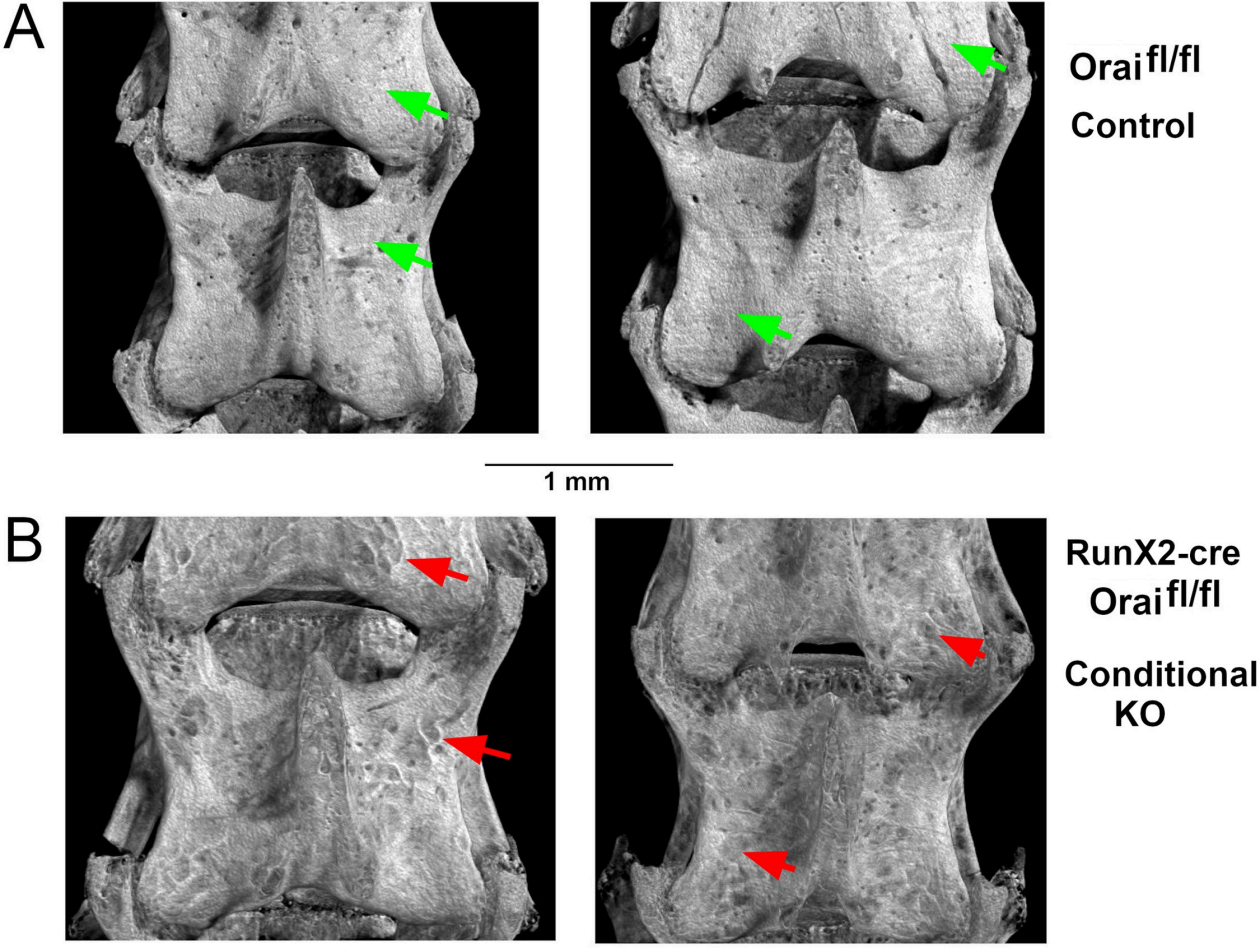
Surface of Orai1^fl/fl^ and Orai1^fl/fl^-Runx2cre fourth lumbar vertebrae. Bruker CTvox software-generated three-dimensional images of vertebrae reconstructed from microCT scans at 5 μm resolution. All animals were homozygous for floxed Orai1; the conditional knockouts (lower panels) are Runx2-cre positive. **A.** Representative vertebrae from control Orai1^fl/fl^ animals without cre recombinase, the control group. Apart from sites of blood vessel entry, the surface of the bone is smooth (green arrows), typical for mice at 16 weeks of age. **B.** Representative vertebrae from Orai1^fl/fl^-Runx2cre animals, the conditional knock out (cKO). In contrast to the control vertebrae, the bone surface appears irregular (red arrows) with patchy darker areas representing regions of reduced bone.

**Fig 2:**
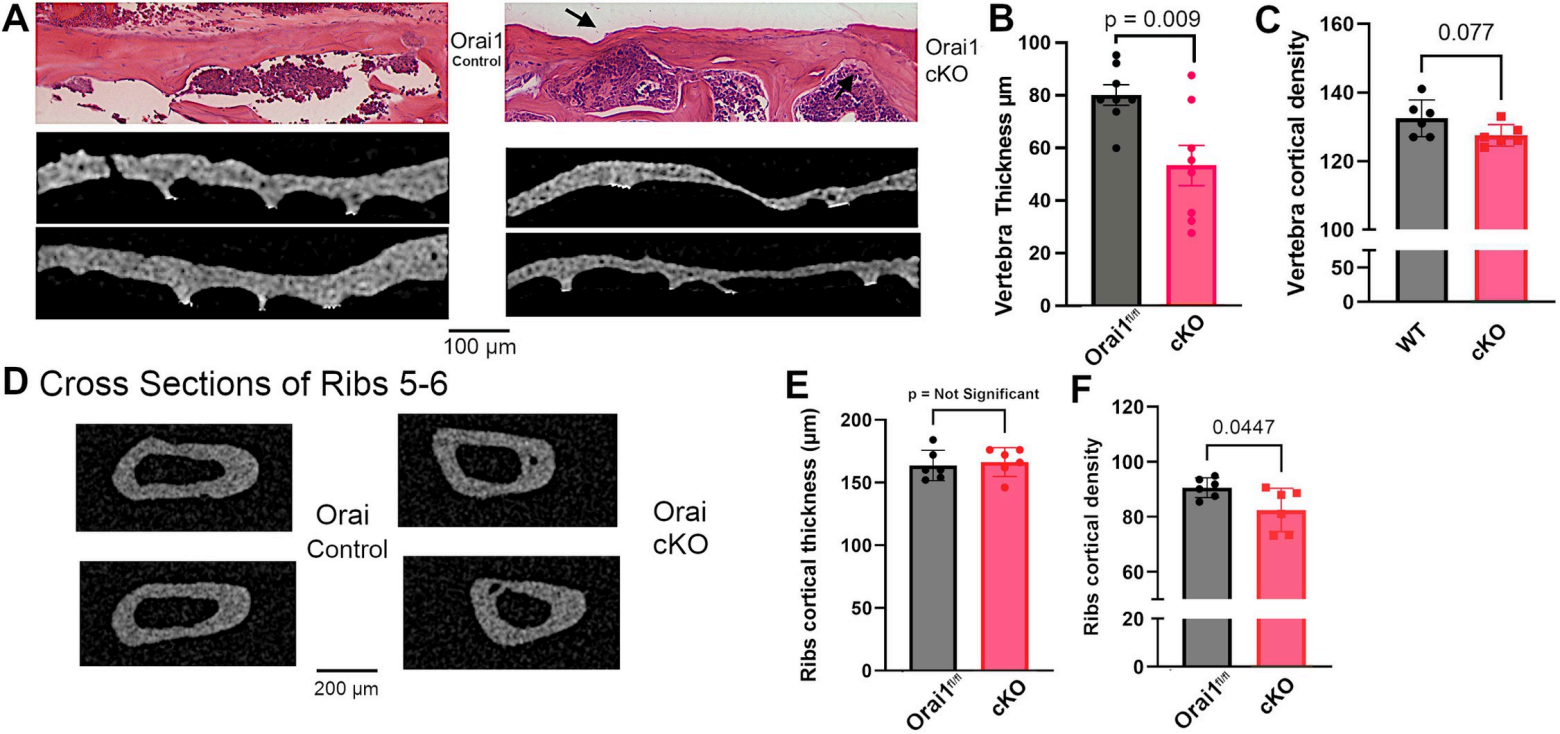
Cross sections of vertebral cortex from Orai1^fl/fl^ controls versus Orai1^fl/fl^-Runx2-cre conditional KO (cKO) animals and ribs 5–6 of Orai1^fl/fl^ versus cKO animals. A. Wild type cortex with typical smooth bone showed relatively uniform thickness compared with Orai1^fl/fl^-Runx2cre (cKO) animals. Images of H&E stained histologic sections from upper lumbar vertebrae (top panels) had irregularly thinned regions (arrow) but no other distinguishing features). Images 1 mm wide; images of microCT scans of lower lumbar vertebrae (middle and lower panels) 1.4 mm wide, excluding trabecular bone for clarity; for trabecular structure see Fig 3. This is in keeping with the appearance of the bone surface in the three-dimensional reconstructions (see Fig 1). B. Cortical bone thickness, though variable, is reduced on average in Orai1^fl/fl^-Runx2cre (Orai1 cKO) animals (p = 0.009, N = 8). C. Cortical bone density by integrating mineral in 8 bit images. A difference comparing 6 measurements, at p = 0.08, was not significant, and the difference was only ~ 2% of the total. D. Cross sections of ribs 5 and 6 compared in Orai1^fl/fl^ and cKO animals. Surfaces were uniformly smooth, in contrast to vertebral cortex (A). E. In contrast to vertebral bone, rib cortical bone thickness did not vary. F. In rib cortical bone a difference between Orai1^fl/fl^ and cKO animals was barely significant, p = 0.04, and difference was < 5%.

Orai1^fl/fl^-Runx2-cre conditional knockout mice revealed no gross differences in size, health or behavior compared to controls. Histo-morphometric studies of vertebrae were performed for detailed assessment of the bone. Wild type vertebrae had typical morphology (examples are shown, Fig 1A), while Orai1^fl/fl^-Runx2-cre vertebrae showed a patchy reduction in cortical bone (examples are shown, Fig 1B). To better understand the basis for this distinct pock-marked appearance in Orai1^fl/fl^-Runx2-cre animals (compare red arrows on Fig 1B with green arrows on Fig 1A), cortical bone was examined in cross section. The appearance of the wild type cortex was unremarkable, but cortical bone in Orai1^fl/fl^-Runx2-cre animals showed irregular thinning (Fig 2A). This lack of regularity is unusual but was observed in three of three Orai1^fl/fl^-Runx2-cre and none of three wild type animals analyzed. Analysis of measurements of cortical thickness, in microns at 300 μm intervals, showed that cortical bone was significantly thinned in the Orai1^fl/fl^-Runx2-cre animals (Fig 2B). Cortical bone density was measured, and a small difference was resolved using 6 vertebrae (Fig 2C). We hypothesized that areas of reduced bone might reflect regions in which Orai1 had been deleted by cre-recombination thus impairing bone formation, but that cre-mediated excision was incomplete with areas of normal thickness reflecting preserved bone formation by osteons with Orai1 positive cells. This was further investigated by antibody labeling for Orai1 (see below). For completeness, we also did basic studies of the effect of the RunX2-cre construct on bone mass relative to complete wild type and the Oria1fl/fl RunX2-cre, which showed no difference between the wild type and RunX2-cre construct without the floxed gene but significant difference with the Oria1fl/flRunX2-cre (Supporting Information first page S1 Fig). Professor Tuckermann (see Authors), who made the RunX2-cre construct, previously did comparisons with wild type (i.e., without the RunX2-cre construct), which also showed that the RunX2-cre construct is not the cause of the observed changes in the conditional KO (Supporting Information first page S2 Fig). Note that S1 Fig and S1 in S1 Data are part of a single Supporting Information file appended.

Long bones were used for cell harvesting and were thus not available for comparison. Given the interesting results in the axial skeleton, this would be a useful goal for follow up study. However, the crucial feature of the long bones is extensive cortical bone, and this was studied using the ribs, which were retained. Comparison with rib architecture showed a major difference, with rib surfaces uniformly smooth (Fig 2D) and no difference in cortical thickness was seen with measurements of 6 ribs (Fig 2E). A small difference in rib bone density was resolved, as with vertebral cortex, using 18 measurements. The meaning of this is uncertain (see Discussion).

#### Orai1^fl/fl^ and Orai1^fl/fl^-Runx2-cre animals show differences by static and dynamic histomorphometry

**Fig 3:**
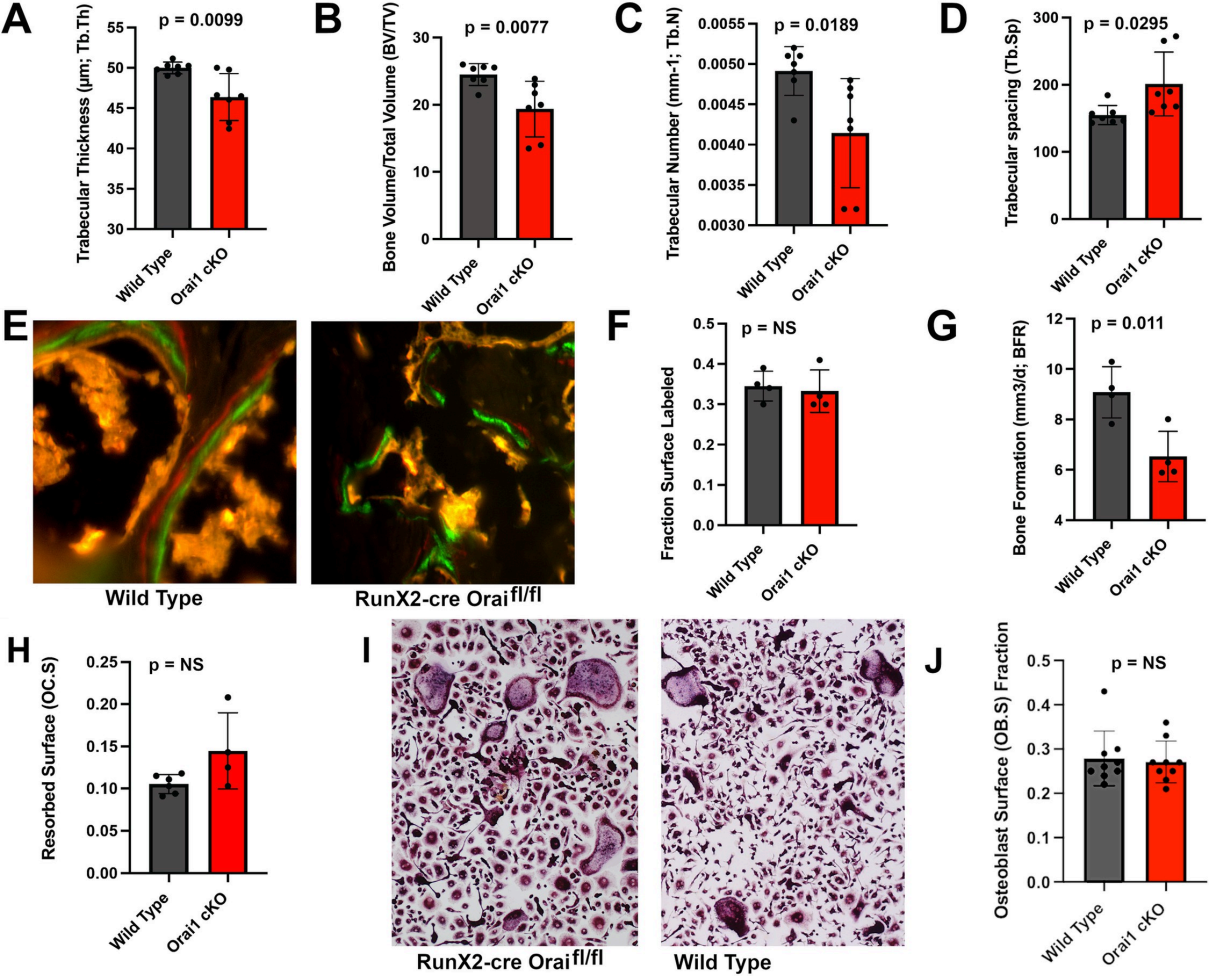
Key features of Orai1^fl/fl^ and conditional KO animals on static and dynamic histomorphometry. A. Bone volume/total volume (BV/TV) of vertebrae. Vertebral bone is significantly reduced (N = 7, p = 0.01) in the Orai1fl/fl-Runx2cre (Orai1 cKO) mice compared to Orai1fl/fl controls without cre recombinase. The greater variability in the conditional-knockout bone is thought to reflect variable cre excision; this variability is also apparent in the vertebral cortex thickness (Fig 2B; see text). **B.** Trabecular thickness is significantly decreased in the Orai1 conditional knockout mice (N = 7, p <0.01). **C.** Trabecular number also decreased significantly in the conditional knockout animals (N = 7, p<0.02). **D.** Trabecular spacing increased in keeping with reduced trabecular number in cKO animals (N = 7, p <0.03) **E.** Example of xylenol orange (red) and calcein (green) fluorescent labels in wild type and conditional KO bones. Interval between double labels represents the bone formation rate in active osteoblasts. The interlabel distance was not statistically different between the control and cKO (not illustrated). See also Fig 3 J below for Ob.S/BS. Fields shown are 100 μm wide. **F.** The proportion of calcein labeled bone surface in wild type and Orai1fl/fl-Runx2cre bone (Orai1 cKO) was not statistically different (N = 4). **G.** The bone formation rate at 4 months of age was significantly reduced in the Orai1fl/fl-Runx2cre mice (Orai1 cKO) compared to Orai1fl/fl controls (N = 4, p = 0.011). **H.** Resorbed surface, 10–15% if bone surface, did not vary significantly between Orai1^fl/fl^ and cKO animals. **I.** Formation of osteoclasts *in vitro* from spleen macrophages with RANKL and CSF-1 did not vary between the Orai1^fl/fl^ and cKO, in contrast to reduced osteoclast formation previously reported in cells from LysM-cre Orai1fl/fl animals [16]. **J.** Osteoblast surface area (bone formation surface) (Ob.S/BS) estimated as cuboidal bone lining cell area [16]; it did not vary mesurably.

Standard histomorphometric parameters for trabecular bone were determined by micro-computed tomography. Results revealed that overall bone mass, measured as bone volume/tissue volume (BV/TV), was reduced in Orai1^fl/fl^-Runx2-cre animals compared to controls (Fig 3A; p = 0.01). The bone mass phenotype was observed at two months and did not change significantly with age up to four months. Mean trabecular thickness was also decreased (Fig 3B; p<0.01) in Orai1^fl/fl^-Runx2-cre bone, as was trabecular number (Fig 3C, p<0.02) in the Orai1 conditional knockouts, and in keeping trabecular spacing increased in the cKO (Fig 3D, p<0.03). Antemortem fluorescent labeling of newly formed bone by injected calcein and xylenol orange was used for dynamic histomorphometry studies. Consistent with the patchy thinner and thicker areas of bone noted above (Figs 1 and 2) suspected to reflect regions of Orai1 negative and positive osteoblasts, we found a patchy distribution of new bone formation with calcein/xylenol orange labeling (Fig 3E) in the Orai1 conditional knock-out animals. This irregular labeling does not fit traditional histomorphometric parameters and was found in none of the controls, but was consistent across the Orai1^fl/fl^-Runx2-cre samples examined. In regions where labeling was intact, dynamic histomorphometric measurements at time of sacrifice showed no difference in bone apposition rate (the interval between double labels, or interlabel distance, not shown) or fraction of labeled bone surface (Fig 3F). Finally, despite the irregularity of the phenotype, the calcein labeling did demonstrate reduction in bone deposition overall in Orai1^fl/fl^-Runx2-cre mice (Fig 3G, p = 0.03). This indicates that bone forming regions in Orai1^fl/fl^-Runx2-cre mice were smaller than in control animals. Given the patchy nature of these effects, we suspect that the contribution of Orai1 to bone formation in affected regions may be variable. Other measurements of osteoblast number including with alkaline phosphatase are possible, and might be useful in future. No evidence of apoptotic cells was seen, another issue for study in future work. To determine if indeed variable Orai1 deletion is the cause of this variable phenotype, we assessed Orai1 deletion further. In addition, we assessed the resorbed area and compared osteoclast formation with RANKL as previously reported in LysM2-cre Orai1^fl/fl^ animals [16] Fig 3H, and examined effect on osteoclast formation *in vitro* (Fig 3I, where no effect was seen. Although the animals studied have floxed Orai1 targeted by RunX2 cre recombinase, indirect effects on resorption are possible and were not studied in detail in this evaluation of Orai1 deletion under the RunX2 promoter, mainly in osteoblasts. However, Fig 3H shows that total resorbed area was 10–15%, indicating that, if there is an increase in resorbed area, it is small. Similarly, there is a possible minor effect on osteoclast differentiation, Fig 3I. Extensive analysis of osteoclasts would require re-derivation of animals, not currently possible, but a subject for future study. For completeness, the area of cuboidal osteoblasts [16] was also measured (Ob.S/BS) Fig 3J. It did not vary significantly between the control and cKO; it also did not vary significantly from area labeled with calcein and Xylenol orange (Fig 3E).

### Variable deletion of osteoblast flox/flox Orai1 by the Runx2-cre promoter

**Fig 4:**
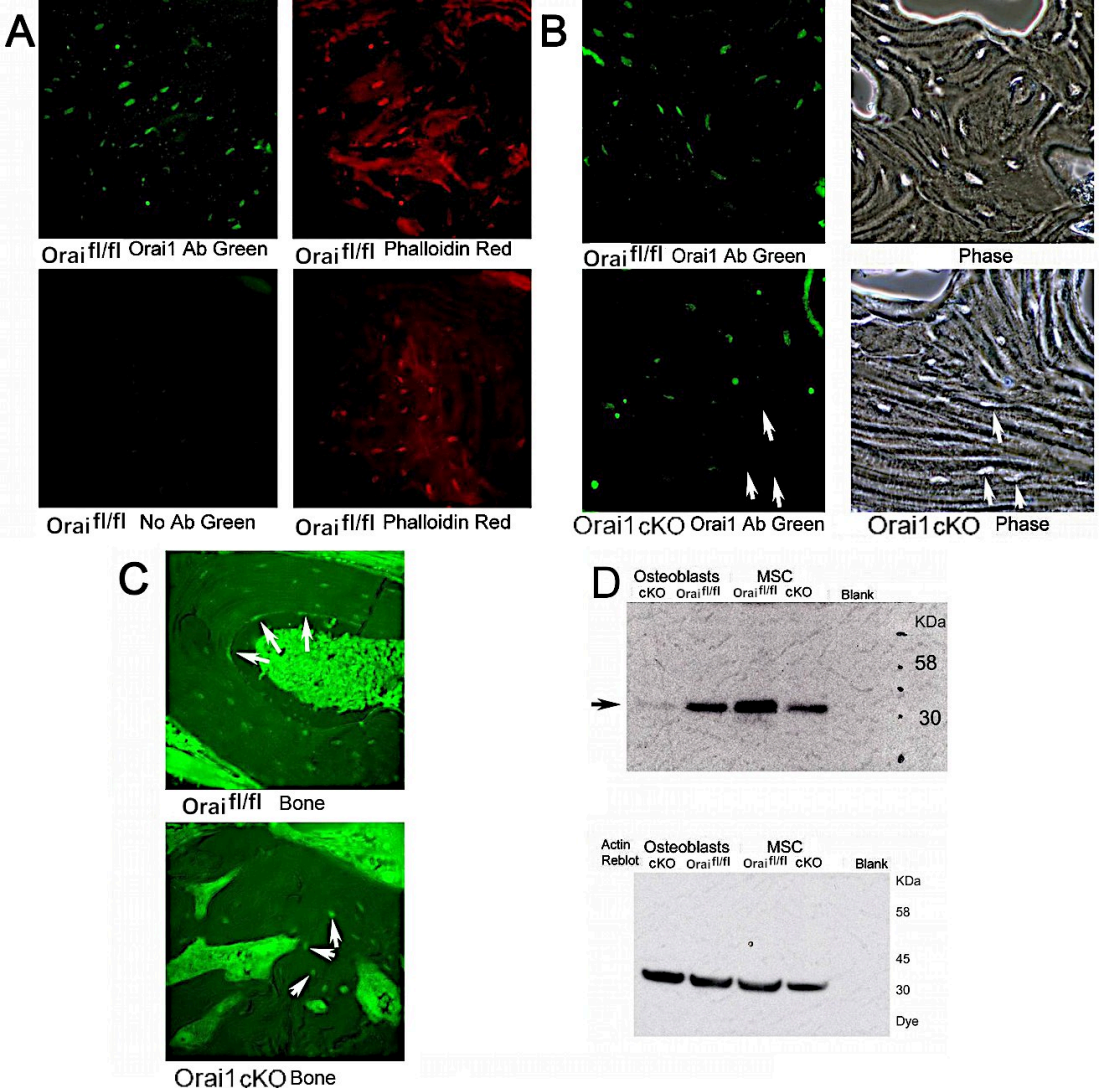
Variable efficacy of Runx2-cre in deleting flox/flox Orai1 in osteoblasts in bone sections. It is often assumed that promoter-cre constructs uniformly delete the target in all cells of an organ. Recent findings suggest this is sometimes not the case (see Text). We tested this by antibody labeling of Orai1fl/fl and Orai1fl/fl-Runx2cre bone. **A.** Rabbit antibody detects Orai1 in osteoblasts (upper left panel). Absence of antibody eliminates labeling (lower left panel). All sections are from one wild type animal. Left and right panels are of the same section. Osteoblasts are shown independently with phalloidin rhodamine (right panels). Fields are 200 μm across. **B.** In the upper panels, in an Orai1fl/fl animal, osteoblasts are shown with the antibody at high power (fields 200 microns wide) and in phase of the same field (right). In the lower panels, the same field of a conditional KO animal (Orai1fl/fl-Runx2cre). Note that some conditional KO cells (Orai1fl/fl-Runx2cre) do not label (arrows, phase and antibody label, lower panels). **C.** Lower power fields, 350 μm, of Orai1 labeled osteoblasts (surrounding tissue fluorescence is an artifact) in Orai1fl/fl bone (top) and Orai1fl/fl-Runx2cre conditional KO bone (bottom). Osteoblasts in the wild type label strongly, including surface cells of the bone (arrows, top frame). Some, but not all, of the osteocytes in the conditional KO label (arrows, bottom panel). **D.** Western blot for Orai1 in cells expressing or not expressing the protein (See Fig 5). Thirty-five μg loads of cell protein from the isolates indicated were run on denaturing SDS-PAGE and blotted. This blot using the very specific Alomone antibody shows a trace of Orai1 even in the osteoblast cKO preparation (left lane) and reduced, but not absent, Orai1 in the MSC cKO (right lane) is shown in the upper panel, in each case relative to Orai1fl/fl controls. The beta actin re-blot (lower panel) confirms similar protein loads. Densitometry showed that labeling in the osteoblast cKO (left lane) relative to Orai1^fl/fl^ was reduced by 93%.

**Fig 5:**
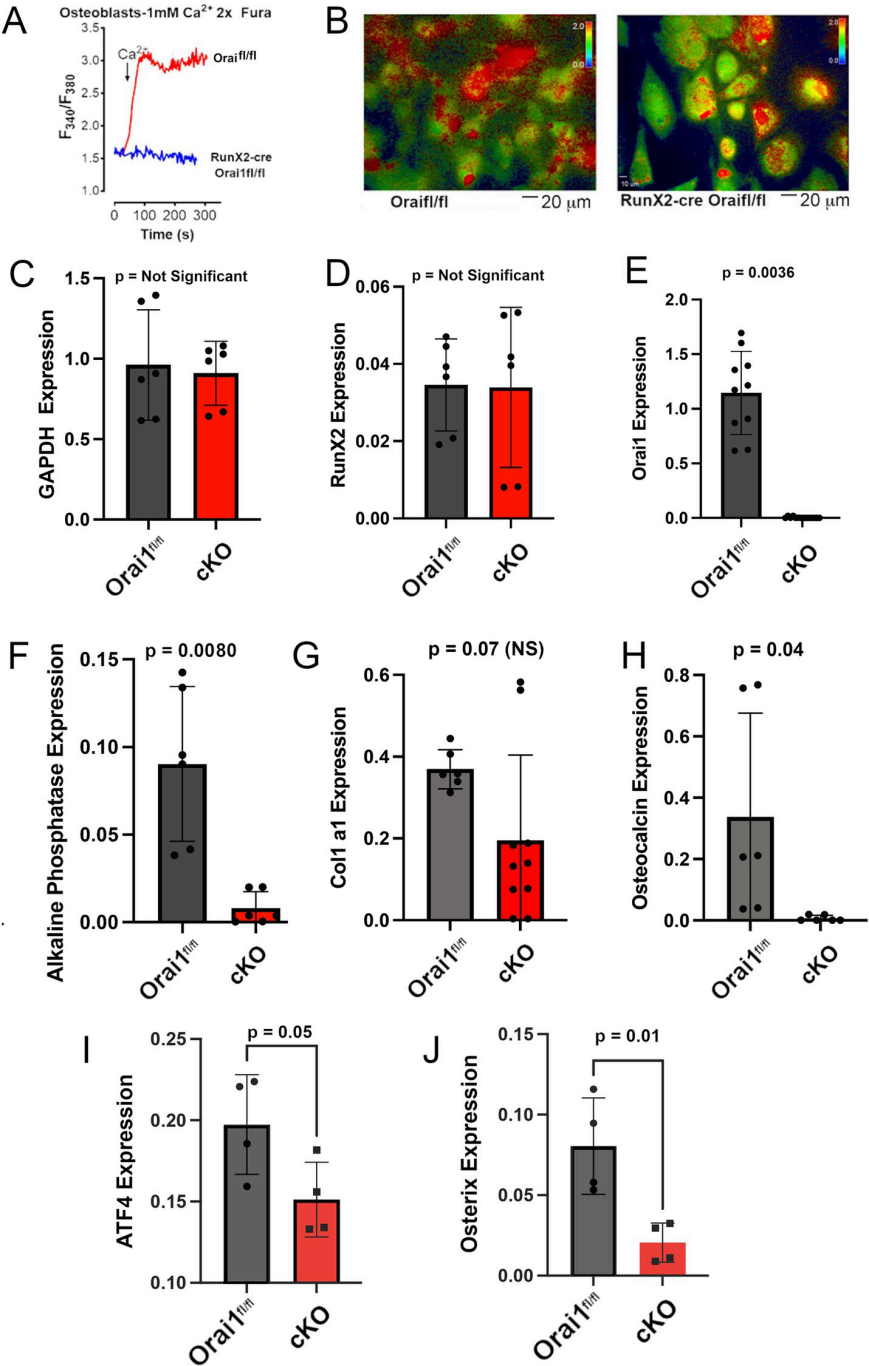
Calcium activated calcium release in cells from Orai^fl/fl^ control and Runx2cre-Orai^fl/fl^ mice and the effect of conditional KO of Orai1 on osteoblasts mRNAs in cells differentiated for one week from MSCs in osteoblast medium. **A.** Intracellular calcium was measured by Fura2 [26] (top panel). Cells were treated with thapsigargin in the absence of extracellular calcium. Only the wild type cells show calcium entry upon extracellular calcium repletion. **B.** Examples of Fura2 signals from control and conditional KO cells, with false color reflecting calcium concentration in cells (scale is shown in upper right corner of each panel). A scale bar for size for the photomicrographs is shown below the bottom panel. **C.** GAPDH expression by PCR in Orai1^fl/fl^ and cKO cells. Differences were not significant. **D.** Expression of RunX2 in in Orai1^fl/fl^ and cKO cells. Differences were also not significant, indicating that recombination with floxed Orai1 was not due to changes in RunX2 transcription. **E.** Expression of Orai1 mRNA was greatly and significantly reduced in cKO cells selected for lack of Orai1 expression (see Fig 5D). **F.** Expression of Alkaline phosphatase was greatly reduced (but not completely absent), in keeping with cellular expression of alkaline phosphatase shown below in Fig 6B. **G.** Expression of Col I a1 was reduced but had high variability and did not reach significance. **H.** Expression of osteocalcin was significantly reduced in the cKO cells. **I.** Expression of osterix (SP7) was significantly reduced in the cKO cells. **J.** Expression of ATF4 (CREB2) was significantly reduced in the cKO cells.

**Fig 6:**
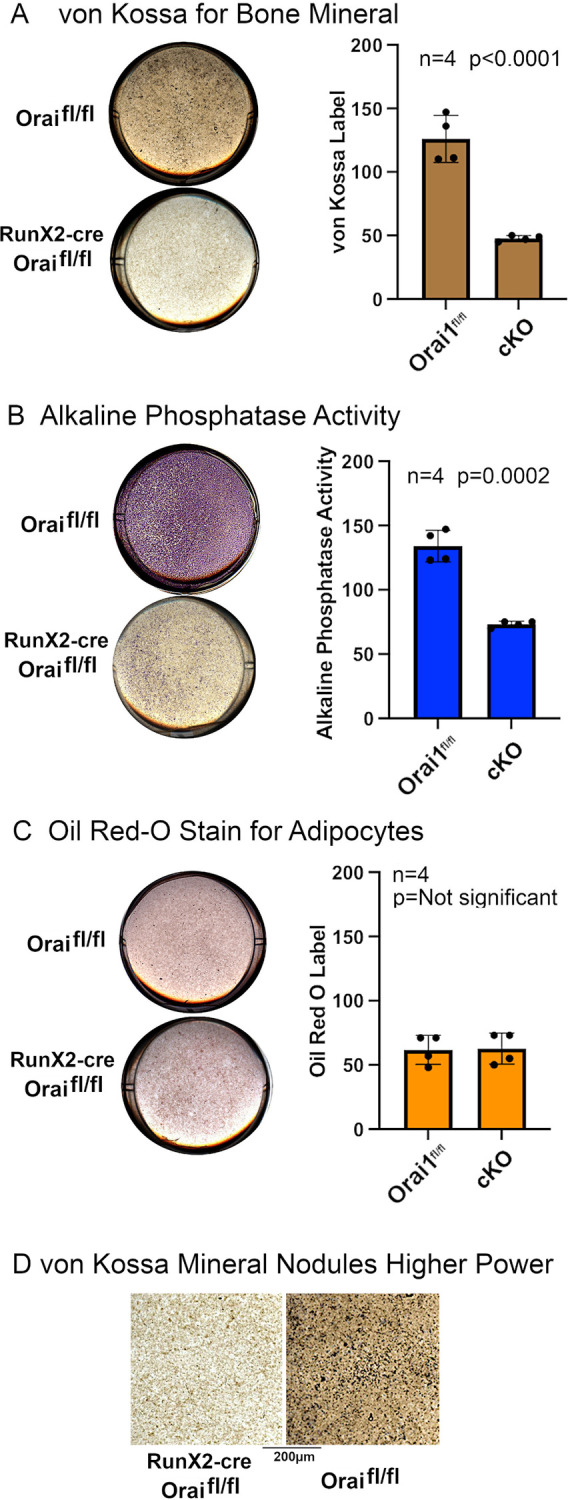
Elimination of Orai1 results in profoundly reduces OB differentiation and mineralization from OB precursors. Osteoblasts isolated as described in the methods section from control Orai1^fl/fl^ or Runx2-cre floxed (Orai1^fl/fl^-Runx2-cre) conditional knock-out animals, incubated for two weeks in osteoblast mineralization medium and analyzed by histologic staining. Each well illustrated is 2 cm across. Note that the cell culture data show a (pure) knockout phenotype, while the in vivo data are of a mosaic, so the larger changes in the Orai1^fl/fl^-Runx2-cre are expected relative to *in vivo* data. **A.** Von Kossa staining for mineral. Wild type cells made mineral nodules, but there were only rare and small nodules in Orai1 knockout cell cultures. Representative culture wells for control (Orai1^fl/fl^) and conditional knockout (cKO Orai1^fl/f^ with RunX2-cre) cells are shown on the left. Staining was quantified for four samples of each genotype. Mineralized matrix production appeared significantly reduced in cultures of Orai1-deficient osteoblasts (p < 0.0001, N = 4).**B.** Alkaline phosphatase activity. Representative cultures are shown on the left. Results quantified from four samples of each genotype are shown on the right. Wild type cells produced much more alkaline phosphatase, with small nodules of high activity as in the silver stain for mineral (A), there was uniformly less alkaline phosphatase in Orai1 null cells (p = 0.0002, N = 4). **C.** Oil red O staining for adipocytes. Representative cultures are shown on the left. Staining was quantified for four samples of each genotype with results shown on the right. There was no evidence of increased adipogenic differentiation in Orai1 null cells, with minimal labeling in cultures of either genotype. This applies only to osteoblast medium, and it is possible that strong adipocyte induction might show effects on adipocytes (see Discussion). **D.** Von Kossa staining of Orai1^fl/fl^ and cKO cells at 2.5 times the magnification shown in (A). The individual clusters of labeled cells in the Orai1^fl/fl^ represent nodules of bone forming cells; these are greatly reduced in the cKO.

Incomplete deletion by Cre has been demonstrated in many different mouse models (see Discussion), although we found the apparent mosaic phenotype of this mouse striking. To demonstrate variable deletion of Orai1 within bone cells, we did Orai1 antibody antibody labeling in wild type and Orai1^fl/fl^-Runx2-cre bone sections (Fig 4). No-added-antibody control for specificity of labeling is shown (Fig 4A, bottom images). While all Orai1^fl/fl^ bone cells expressed Orai1 (Fig 4A), there were patches of Orai1-negative (arrows) as well as Orai1-positive osteocytes in Orai1^fl/fl^-Runx2-cre mice (Fig 4B and 4C, lower panels). Western blot of cultured osteoblasts and MSCs showed incomplete Orai1 deletion of Orai1^fl/fl^-Runx2-cre osteoblasts (Fig 4D). This is consistent with deletion of Orai1 in osteoblasts from Orai1^fl/fl^-Runx2-cre mice. To determine the functional implications of Orai1 deletion in osteoblasts, *in vitro* cell cultures, negative for Orai1 by PCR, in mineralization medium, were used, Figs 5 and 6.

### Effect of orai1 deletion on store-operated calcium entry

MSCs were collected from Orai1fl/fl and Orai1^fl/fl^-Runx2-cre mice before differentiating into osteoblasts *in vitro* and measuring store-operated calcium entry as described [16]. Cells were treated with thapsigargin in the absence of extracellular calcium to deplete the endoplasmic reticulum of calcium and initiate the store-operated calcium response. While a typical store-operated calcium response was observed in Orai1-expressing osteoblasts, loss of Orai1 completely eliminated store-operated calcium entry (Fig 5A), with images of Fura2 labeled cells shown (Fig 5B). These observations show that Orai1 is required for store-operated calcium entry in osteoblasts. Further, real-time PCR studies [12] were used to compare GAPDH, RunX2, Orai1, Alkaline Phosphatase, Col1 and osteocalcin mRNA in osteoblasts differentiated for one week from Orai1^fl/fl^ versus Orai1 negative MSCs (Fig 5C–5H). There was no difference in GAPDH or RunX2 expression, in keeping with normal expression of the RunX2-cre signal, but Alkaline phosphatase and Orai1 were both greatly reduced, in keeping with Fig 5B and reduced osteoblast’s differentiation in the same cells at two weeks in osteoblast differentiation medium, shown in Fig 6. Type I collagen expression was decreased but not significantly due to variability, while the more specific osteocalcin (bone gla protein) was significantly reduced (Fig 5G and 5H). Because RunX2 was not decreased in conditional KO cells (Fig 5D), we ran PCR in control and cKO cells for two other transcription factors important for promotion of osteoblast differentiation: osterix and ATF4 (Fig 5I and 5J). Both of these were significantly reduced, in keeping with the expectation that poor osteoblast differentiation might be related to reduced activity of these factors.

### Orai1^fl/fl^-Runx2-cre cells showed severe defects in osteoblast differentiation

We compare osteoblast differentiation using wild type cells versus cells from conditional knockouts in which absence of Orai1 was confirmed by PCR. Cells were cultured for two weeks in mineralization medium with 2-glycerol phosphate and ascorbate. Mineral deposition was detected using silver nitrate (von Kossa); mineralization was prominent in cultures of wild type cells, but markedly reduced for Orai1^fl/fl^-Runx2-cre osteoblasts (Fig 6A). Additionally, alkaline phosphatase positivity in the conditional KO was significantly decreased relative to wild type (Fig 6B). Note that clusters of alkaline phosphatase-producing cells are present, but these are greatly reduced, in keeping with alkaline phosphatase mRNA shown in Fig 5F above. Reduced osteoblastic differentiation was reported to correlate with increased adipocytes in mice fed a Western diet [27]. However, no Orai1-dependent differences in adipogenic differentiation were identified by Oil red O staining in these culture conditions (Fig 6C), suggesting that Orai1 expression did not favor osteoblast-adipocyte conversion. For definitive evaluation of the role of Orai1 further studies using adipogenic culture conditions will be needed (see Discussion). The mineral labeling is shown at higher magnification in Fig 6D, where nodules of mineralizing cells are seen in Orai1^fl/fl^ and greatly reduced in the cKO.

## Discussion

We show that Orai1 serves an essential, although not absolute, cell-intrinsic function in osteoblast differentiation. Interpretation is complicated by incomplete deletion of Orai1, resulting in animals exhibiting an intriguing mosaic phenotype. Interestingly, complete deletion of glucocorticoid genes early in osteoblast differentiation has been reported in Runx2-cre mice [20]. While the reasons for this difference are not clear; epigenetic differences in the status of the Orai1 gene amongst different osteoblasts represent one possible explanation; silencing of genes due to histone methylation have been shown to interfere with cre recombinases in prior study [28]. While this possibility has not been established for the Runx2-cre floxed Orai1 combination, it would be consistent with the findings reported here and a subject for future study. In keeping, other studies have shown incomplete cre-mediated excision [29, 30].

Global analysis of bone density showed significant but subtle differences. We feel that this likely reflects the incomplete deletion of Orai1 *in vivo*. However, *in vitro* bone differentiation from Orai1^-/-^ cells caused profound reductions in osteoblast differentiation. This reflects that cells used were either wild type or confirmed Orai1^-/-^, while *in vivo* a patchwork of normal and Orai1^-/-^ cells occurs.

Other very interesting findings included that in peripheral bone, the distinct non-uniformity of bone cortex in the vertebrae did not occur (Fig 2A versus 2D). In future work, direct study of the tibia or femur will be done to extend this work, but animals are at present not available and re-derivation is not practical. However, the effect on cortical bone indicates that dependency of bone formation on Orai1 may vary with skeletal location. Additionally, Runx2 is expressed in growth plate chondrocytes and detailed analysis of effects on the growth plate would best be done in the long bone growth plates, in future work.

The bone mass phenotype (Fig 3) was observed at ~ four months. A topic of potential interest is effect on bone mass of age at longer times, a topic for future work with the conditional knockout model. Both axial (vertebral) and peripheral bone had small reductions of bone density, a few percent and in vertebral bone not significant (Fig 2C and 2F), but clearly where bone did mineralize, effects of Orai1 conditional KO were minor. This is in keeping with lack of effect on inter-label fluorescent mineral formation assays shown in Fig 3. Other measurements of osteoblast activity such as alkaline phosphatase might be useful in future work when animals are re-derived, as would be direct assays for apoptosis, but are impractical in this initial study. The fluorescent labeling of new bone mineral is the best, and quantitative, determination of mineral formation. In contrast to the effect of LysM2-cre driven Orai1^fl/fl^ on bone resorption [16], total resorbed space was 10–15%, indicating that if there is an increase in resorbed area it is small (Fig 3H). There is also a possible minor effect on osteoclast differentiation, Fig 3I. Detailed study of this interesting factor would require re-derivation of animals, and is deferred to future study.

Further, while Runx2 is crucial for the commitment of mesenchymal stem cells to the osteoblast lineage, Runx2 expression is down-regulated at later stages [31]. While there is convincing evidence that Orai1 is involved in osteoblast differentiation and function in the current study and in [32], the uncertain status of Orai1 in osteoblast precursors combined with the temporary nature of Runx2 expression *in vivo* may contribute to the mosaic phenotype in this model. As expected, the conditional KO reduced Orai1 and alkaline phosphatase mRNA expression, and decreased collagen expression (but not significantly) while significantly reducing osteocalcin (bone gla protein) (Fig 5E–5I). RunX2 did not change significantly in these cells (Fig 5D), likely because RunX2 drives the knock-out of Orai1 and thus the inhibitory effects of loss of Orai1 occur either subsequent to, or in pathways parallel to, RunX2. Because RunX2 did not change, we examined ATF4 and osterix, two other important regulators of osteoblast differentiation, by real-time PCR in Orai1fl/fl and cKO cells (Fig 5I and 5J); expression of these transcription factors was, as expected, reduced in cKO cells, consistent with impairment of osteoblastic differentiation. Effects on bone differentiation in vitro (Fig 6) were consistent with the PCR findings (Fig 5) and did not show adipocytes, although only osteoblast differentiation conditions were used. Effect of direct stimulation of adipocytes should be studied before it is concluded that adipocytes are not affected by Orai1 expression. Specifically, direct stimulation by insulin, isobutylmethylxanthine and cortisol are needed before it is concluded that adipocytes are not affected by the Orai1 conditional knockout. Oher assays could be useful in future work that were not included this initial study of the model. As an example, serum chemistry for alkaline phosphatase and bone turnover markers may also be informative.

The present work using non-transformed cells indicates unequivocally an essential role of Orai1 in bone differentiation. When Orai1 is eliminated in pre-osteoblastic cells, limited osteoblast differentiation occurs, with remarkable variability of osteoblast-related differentiation. The ability of osteoblasts to form mineralizing nodules is completely abrogated in Orai1-null osteoblasts. This is consistent with the necessity of store-operated calcium entry for bone differentiation. This further suggests that the formation of advanced osteoblast-related structures [33] is impossible without store-operated calcium entry. There are a many studies indicating importance for calcium signals in bone formation, e.g., [34–36], but no studies previously demonstrated *in vivo* the requirement for the Orai1 calcium-released activated calcium channel in normal osteoblast differentiation. Our work is cell specific, unlike general Orai1 knockout studies [12, 32], in which bone degrading, bone forming, and potentially other cell types were affected. In truly Orai1 negative osteoblasts, bone differentiation is severely compromised.

## Supporting information

S1 Fig In preliminary work we also compared RunX2-cre to complete wild type (no cre). There was, as expected, no difference; the cre/floxed animals had a significant decrease in bone volume/total volume, matching RunX2 cre versus RunX2 cre with floxed Orai (See also Fig 3A). Generally, comparison of complete wild type and cre recombinase only is not done: Without a floxed gene the cre has no activity.
(TIF)

S2 Fig In other work from the project by Tuckermann et al., studying conditional KO of menin [37], not previously published, RunX2cre-floxed expression was compared to complete wild type. For each panel, density and SD, N = 4, are shown. No effect of the wild type recapitulating the cre-floxed phenotype occurred. The left panel shows the floxed gene (menin1, Men1) only, the second panel the conditional KO (Men1RunX2cre), the third panel wild type, and the fourth panel RunX2 cre only. Only the conditional KO showed significant effect (p<0.01, **).
(TIF)

S1 Data (PDF)
