# Supplementary figures and images for: The calcium channel Orai1 is required for osteoblast development: Studies in a chimeric mouse with variable in vivo Runx-cre deletion of Orai-1
Source: PLoS One. 2023 May 11;18(5):e0264596. doi: 10.1371/journal.pone.0264596 (PMC10174572; doi:10.1371/journal.pone.0264596)

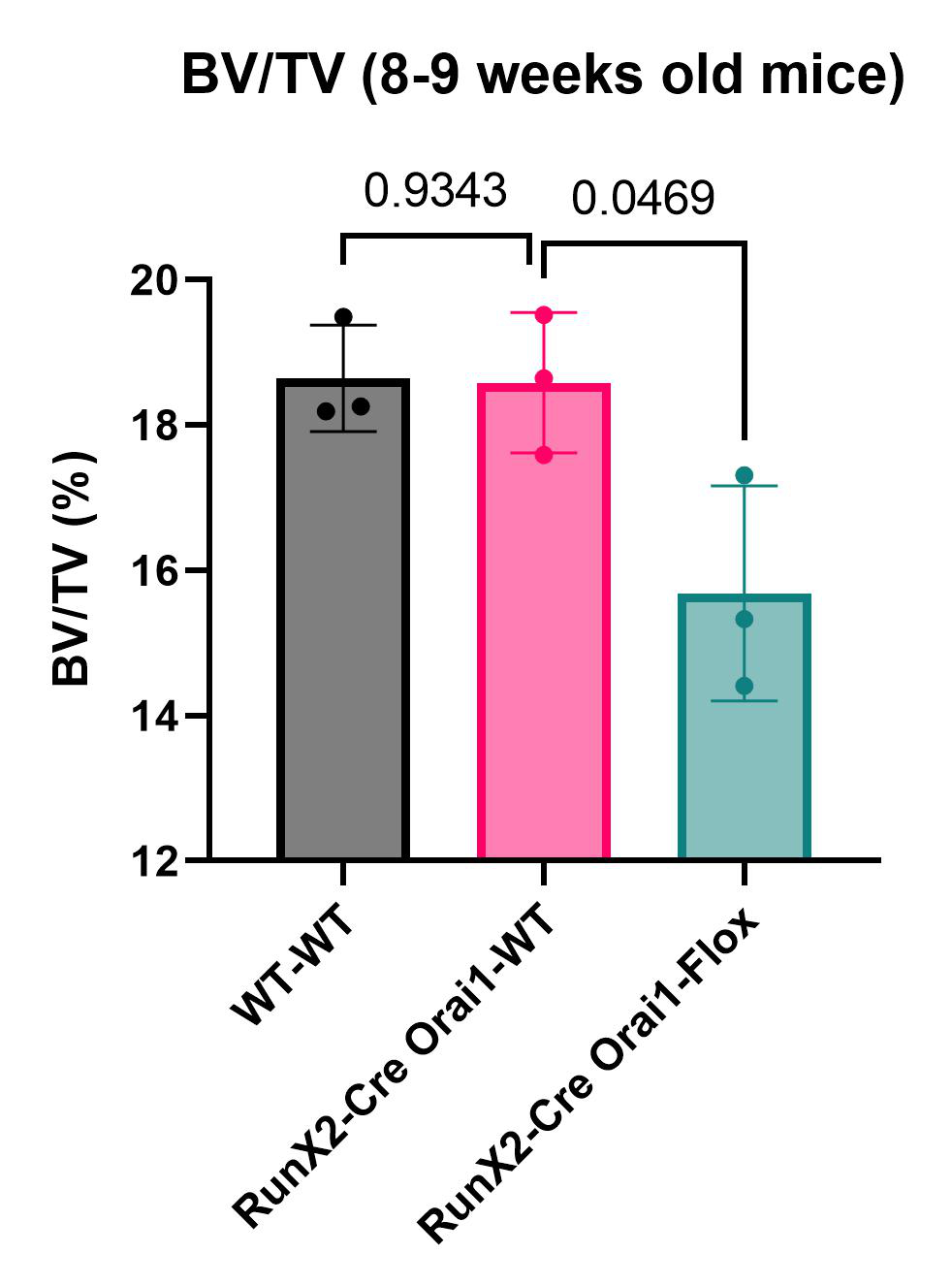

Supplement: S1 Fig — There was, as expected, no difference; the cre/floxed animals had a significant decrease in bone volume/total volume, matching RunX2 cre versus RunX2 cre with floxed Orai (See also Fig 3A). Generally, comparison of complete wild type and cre recombinase only is not done: Without a floxed gene the cre has no activity. (TIF) [file pone.0264596.s001.tif]

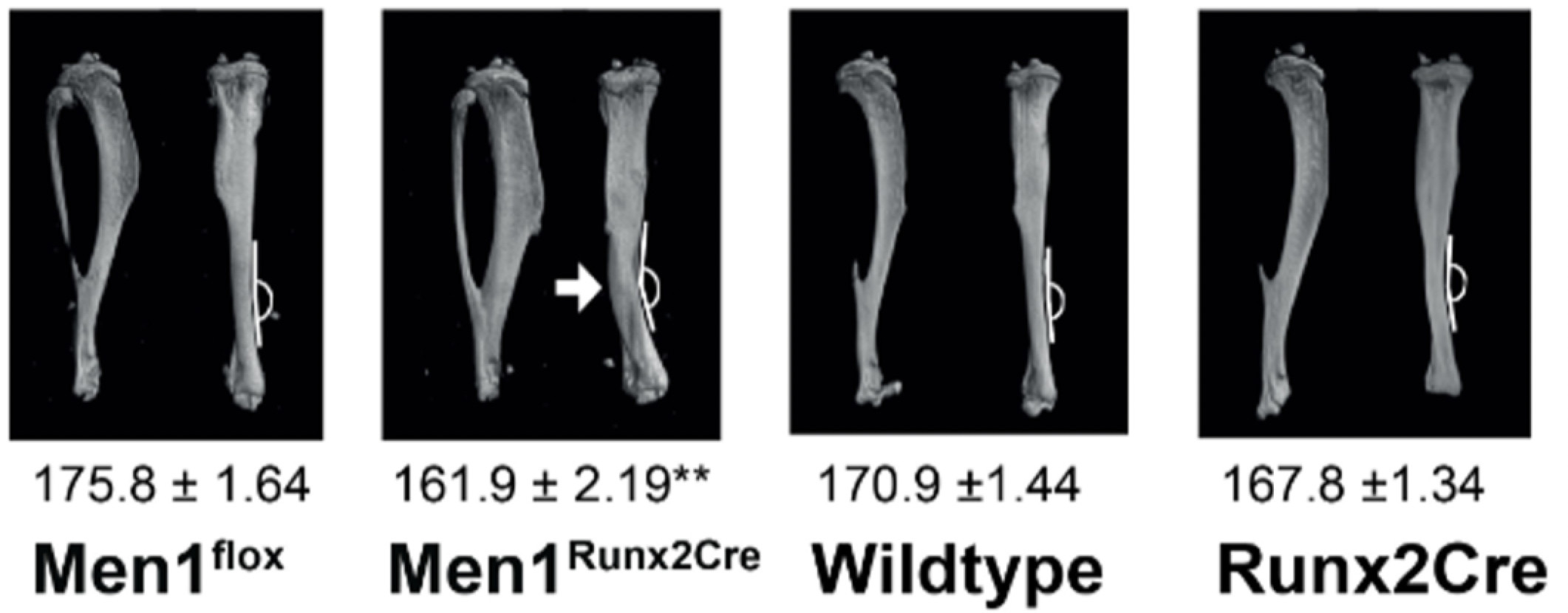

Supplement: S2 Fig — For each panel, density and SD, N = 4, are shown. No effect of the wild type recapitulating the cre-floxed phenotype occurred. The left panel shows the floxed gene (menin1, Men1) only, the second panel the conditional KO (Men1RunX2cre), the third panel wild type, and the fourth panel RunX2 cre only. Only the conditional KO showed significant effect (p<0.01, **). (TIF) [file pone.0264596.s002.tif]
