# Supplementary material for: The calcium channel Orai1 is required for osteoblast development: Studies in a chimeric mouse with variable in vivo Runx-cre deletion of Orai-1
Source: PLoS One. 2023 May 11;18(5):e0264596. doi: 10.1371/journal.pone.0264596 (PMC10174572; doi:10.1371/journal.pone.0264596)
Supplement: S1 Data — (PDF) [file pone.0264596.s003.pdf]

Supporting information for PONE-D-22-03926

1) Project tracking for all animals and preparations. This includes names and genotypes of all animals used. Genotyping, as specified in the Methods, was done independently by Jax Labs in a contract arrangement.

A. Original mouse names and breeding information.

#Backcrossed	Mice ID	Gender	Orai1-fl	runx2-cre	Orai1	Birth Date	Parents M/F					Sacrifice
R9	Rx72A	F	**mut/mut**	**wt/mut**	-/-	7/20/16	Rx61A	Rx62A	Rx68A	Orai1KO		1/25/17
R9	Rx72D	F	**mut/mut**	**wt/mut**	-/-	7/20/16	Rx61A	Rx62A	Rx68A	Orai1KO		1/25/16
R9	Rx90A	M	**mut/mut**	**wt/mut**	-/-	10/25/16	Rx61A	Rx87A	Rx72D	Orai1KO		2/15/16
R9	Rx90C	M	**mut/mut**	**wt/mut**	-/-	10/25/16	Rx61A	Rx87A	Rx72D	Orai1KO		2/15/16
R10	Rx86B	F	**mut/mut**	wt/wt	+/+	9/12/16	Rx69B	Rx70D			House with Rx83E, Rx87B on February 21	
R9	Rx90A	M	**mut/mut**	wt/wt	+/+	11/18/16	Rx82B	Rx83E	Rx87B		calcein injections for sacrifice March 13	
R9	Rx83E	F	**mut/mut**	wt/wt	+/+	9/12/16	OC83B	Rx66B	Rx66C		calcein injections for sacrifice March 22	
R9	Rx87B	F	**mut/mut**	wt/wt	+/+	9/14/16	Rx67B	Rx66A			calcein injections for sacrifice March 22	
R9	Rx73A	M	**mut/mut**	**wt/mut**	-/-	8/3/16	Rx67B	Rx66A	Rx68D	Orai1KO		
R9	Rx87A	F	**mut/mut**	**wt/mut**	-/-	9/14/16	Rx67B	Rx66A		Orai1KO	calcein injections for sacrifice March 29	
R9	Rx87C	F	**mut/mut**	**wt/mut**	-/-	9/14/16	Rx67B	Rx66A		Orai1KO	calcein injections for sacrifice March 29	
R9	Rx87D	F	**mut/mut**	**wt/mut**	-/-	9/14/16	Rx67B	Rx66A		Orai1KO	calcein injections for sacrifice March 29	
R9	Rx90B	M	**mut/mut**	wt/wt	+/+	11/18/16	Rx82B	Rx83E	Rx87B		calcein injections for sacrifice March 22	
R9	Rx91D	F	**mut/mut**	wt/wt	+/+	11/18/16	Rx82B	Rx83E	Rx87B		calcein injections for sacrifice March 13	
R10	Rx92A	F	**mut/mut**	wt/wt	+/+	11/21/16	Rx85E	Rx86B			calcein injections for sacrifice March 15	
R10	Rx92B	F	**mut/mut**	wt/wt	+/+	11/21/16	Rx85E	Rx86B			calcein injections for sacrifice March 15	
R9	Rx93A	M	**mut/mut**	**wt/mut**	-/-	12/10/16	Rx73A	Rx72A	Rx87D	Orai1KO	calcein injections for sacrifice April 10	
R9	Rx93B	M	**mut/mut**	**mut/mut**	-/-	12/10/16	Rx73A	Rx72A	Rx87D	Orai1KO	calcein injections for sacrifice April 10	

R9	Rx94A	F	**mut/mut**	**wt/mut**	-/-	12/10/16	Rx73A	Rx72A	Rx87D	Orai1KO	calcein injections for sacrifice April 11
R9	Rx94B	F	**mut/mut**	**wt/mut**	-/-	12/10/16	Rx73A	Rx72A	Rx87D	Orai1KO	calcein injections for sacrifice April 11
R9	Rx96D	F	**mut/mut**	**wt/mut**	-/-	12/11/16	Rx90C	Rx87A	Rx72D	Orai1KO	calcein injections for sacrifice April 12
R9	Rx95A	M	**mut/mut**	**mut/mut**	-/-	12/11/16	Rx90C	Rx87A	Rx72D	Orai1KO	calcein injections for sacrifice April 12
R9	Rx95B	M	**mut/mut**	**wt/mut**	-/-	12/11/16	Rx90C	Rx87A	Rx72D	Orai1KO	calcein injections for sacrifice April 12
R9	Rx95C	M	**mut/mut**	**wt/mut**	-/-	12/11/16	Rx90C	Rx87A	Rx72D	Orai1KO	calcein injections for sacrifice April 13
R9	Rx95D	M	**mut/mut**	**wt/mut**	-/-	12/11/16	Rx90C	Rx87A	Rx72D	Orai1KO	calcein injections for sacrifice April 13
R9	Rx95E	M	**mut/mut**	**wt/mut**	-/-	12/11/16	Rx90C	Rx87A	Rx72D	Orai1KO	calcein injections for sacrifice April 13
R10	Rx97A	M	**mut/mut**	wt/wt	+/+	12/28/16	Rx85E	Rx86B			calcein injections for sacrifice April 17
R10	Rx97B	M	**mut/mut**	wt/wt	+/+	12/28/16	Rx85E	Rx86B			calcein injections for sacrifice April 17
R10	Rx97C	M	**mut/mut**	wt/wt	+/+	12/28/16	Rx85E	Rx86B			calcein injections for sacrifice April 19
R10	Rx97D	M	**mut/mut**	wt/wt	+/+	12/28/16	Rx85E	Rx86B			calcein injections for sacrifice April 19
R10	Rx97E	M	**mut/mut**	wt/wt	+/+	12/28/16	Rx85E	Rx86B			calcein injections for sacrifice April 19
R9	Rx98A	M	**mut/mut**	wt/wt	+/+	1/10/17	Rx90A	Rx87C			calcein injections for sacrifice May 1
R9	Rx98B	M	**mut/mut**	**mut/mut**	-/-	1/10/17	Rx90A	Rx87C		Orai1KO	calcein injections for sacrifice May 1
R9	Rx98C	M	**mut/mut**	wt/wt	+/+	1/10/17	Rx90A	Rx87C			calcein injections for sacrifice May 1
R9	Rx98D	M	**mut/mut**	**mut/mut**	-/-	1/10/17	Rx90A	Rx87C		Orai1KO	calcein injections for sacrifice May 3
R9	Rx98E	M	**mut/mut**	wt/wt	+/+	1/10/17	Rx90A	Rx87C			calcein injections for sacrifice May 3
R9	Rx99A	F	**mut/mut**	**wt/mut**	-/-	1/10/17	Rx90A	Rx87C		Orai1KO	calcein injections for sacrifice May 4
R9	Rx99B	F	**mut/mut**	wt/wt	+/+	1/10/17	Rx90A	Rx87C			calcein injections for sacrifice May 4
R9	Rx100A	M	**mut/mut**	wt/wt	+/+	1/24/17	Rx90A	Rx83E	Rx87B		calcein injections for sacrifice May 15
R9	Rx100B	M	**mut/mut**	wt/wt	+/+	1/24/17	Rx90A	Rx83E	Rx87B		calcein injections for sacrifice May 15
R9	Rx100C	M	**mut/mut**	wt/wt	+/+	1/24/17	Rx90A	Rx83E	Rx87B		calcein injections for sacrifice May 16
R9	Rx100D	M	**mut/mut**	wt/wt	+/+	1/24/17	Rx90A	Rx83E	Rx87B		calcein injections for sacrifice May 16
R9	Rx101A	F	**mut/mut**	wt/wt	+/+	1/24/17	Rx90A	Rx83E	Rx87B		calcein injections for sacrifice May 17
R9	Rx101B	F	**mut/mut**	wt/wt	+/+	1/24/17	Rx90A	Rx83E	Rx87B		calcein injections for sacrifice May 17
R9	Rx101C	F	**mut/mut**	wt/wt	+/+	1/24/17	Rx90A	Rx83E	Rx87B		calcein injections for sacrifice May 17
R9	Rx102A	M	**mut/mut**	wt/wt	+/+	2/5/17	Rx90C	Rx87C			calcein injections for sacrifice May 31

R9	Rx102B	M	**mut/mut**	wt/wt	+/+	2/5/17 Rx90C	Rx87C		calcein injections for sacrifice May 31
R9	Rx103A	F	**wt/mut**	**wt/mut**	+/+	2/5/17 Rx90C	Rx87C	Kill	
R9	Rx103B	F	**mut/mut**	wt/wt	+/+	2/5/17 Rx90C	Rx87C		calcein injections for sacrifice May 29
R9	Rx103C	F	**wt/mut**	wt/wt	+/+	2/5/17 Rx90C	Rx87C	Kill	
R9	Rx103D	F	**mut/mut**	**wt/mut**	-/-	2/5/17 Rx90C	Rx87C	Orai1KO	calcein injections for sacrifice May 29

B. Mice with genotypes and major assays including micro CT.

	Mice are listed in Order received	Genotype	temple						WVU Initial collection		WVU Genotype	Genoty
Notes*	Mouse ID	Orai1-fl	runx2-cre	Orai1	Sex	Birthdate	Date of sac	Age @ sac	# vials BM	#vials non-adh	other	msc
pooled @ Pitt	14A	wt/wt	**wt/mut**	+/+	M	5/29/14	7/30/14	9 weeks				
pooled @ Pitt	17A	wt/wt	**wt/mut**	+/+	F	6/2/14	7/30/14	9 weeks	?			cre
pooled @ Pitt	17C	wt/wt	**wt/mut**	+/+	F	6/2/14	7/30/14	9 weeks				
pooled @ Pitt	14D	mut/mut	**wt/mut**	-/-	M	5/29/14	7/30/14	9 weeks				
pooled @ Pitt	15B	mut/mut	**wt/mut**	-/-	M	6/4/14	7/30/14	9 weeks	?			flox cre
pooled @ Pitt	15C	mut/mut	**wt/mut**	-/-	M	6/4/14	7/30/14	9 weeks				
	6mo WT	wt/wt	wt/wt	+/+	F	?	11/17/16	6mo	2	0		
	Rx 72A	**mut/mut**	**wt/mut**	-/-	F	7/20/16	1/25/17	27w	1	2		flox-cre
	Rx 72D	**mut/mut**	**wt/mut**	-/-	F	7/20/16	1/25/17	27w	2	2		flox-cre
	Rx 90AKO	mut/mut	**wt/mut**	-/-	M	10/25/16	2/15/17	16w 1d	1	1		NA
	~~Rx 90C~~	mut/mut	**wt/mut**	-/-	M	10/25/16	2/15/17	16w 1d	2	1		
*snow arr. 3/17	Rx 90AZ	mut/mut	wt/wt	+/+	M	11/18/16	3/13/17	16w 3d	2	0		NA
*snow arr. 3/17	Rx 91D	mut/mut	wt/wt	+/+	F	11/18/16	3/13/17	16w 3d	2	0		NA
	Rx 92A	mut/mut	wt/wt	+/+	F	11/21/16	3/15/17	16w 2d	3	0	m-flox-wt	NA
	~~Rx 92B~~	mut/mut	wt/wt	+/+	F	11/21/16	3/15/17	16w 2d	2 or 3	0	m-het flox-cre	

	Rx 90B	mut/mut	wt/wt	+/+	M	11/18/16	3/21/17	17w 4d	2		NA
	Rx 83E	mut/mut	wt/wt	+/+	F	9/12/16	3/21/17	27w 1d	5		flox-wt
	Rx 87B	mut/mut	wt/wt	+/+	F	9/14/16	3/21/17	26w 6d	2		
	Rx 86B	mut/mut	wt/wt	+/+	F	9/12/16	3/29/17	28w 2d	2		NA
	Rx 87A	**mut/mut**	**wt/mut**	-/-	F	9/14/16	3/29/17	28w	2		NA
	Rx 87C	**mut/mut**	**wt/mut**	-/-	F	9/14/16	3/29/17	28w	3		NA
	Rx 87D	**mut/mut**	**wt/mut**	-/-	F	9/14/16	3/29/17	28w	2		NA
	Rx 93A	mut/mut	**wt/mut**	-/-	M	12/10/16	4/10/17	17w 2d	3	m-flox-cre	NA
	Rx 93B	mut/mut	**mut/mut**	-/-	M	12/10/16	4/10/17	17w 2d	3		NA
	Rx 94A	mut/mut	**wt/mut**	-/-	F	12/10/16	4/10/17	17w 2d	2		
	Rx 94B	mut/mut	**wt/mut**	-/-	F	12/10/16	4/10/17	17w 2d	2		NA
	Rx 95A	mut/mut	**mut/mut**	-/-	M	12/11/16	4/11/17	17w 2d	2	m-flox-cre	
	Rx 95B	mut/mut	**wt/mut**	-/-	M	12/11/16	4/11/17	17w 2d	3	m-flox-?wt	
	Rx 95C	mut/mut	**wt/mut**	-/-	M	12/11/16	4/11/17	17w 2d	2	s-?flox-cre	
aka 96A	Rx 96D	mut/mut	**wt/mut**	-/-	F	12/11/16	4/11/17	17w 2d	2		NA
	Rx 95D	mut/mut	**wt/mut**	-/-	M	12/11/16	4/12/17	17w 1d	2	s-?flox-cre	
	Rx 95E	mut/mut	**wt/mut**	-/-	F	12/11/16	4/12/17	17w 1d	2	s-?flox-cre	NA
	Rx 97A	mut/mut	wt/wt	+/+	M	12/28/16	4/17/17	15w 5d	2		
	Rx 97B	mut/mut	wt/wt	+/+	M	12/28/16	4/17/17	15w 5d	2		NA
	Rx 97C	mut/mut	wt/wt	+/+	M	12/28/16	4/19/17	16w	2		
	Rx 97D	mut/mut	wt/wt	+/+	M	12/28/16	4/19/17	16w	2		NA
	Rx 97E	mut/mut	wt/wt	+/+	M	12/28/16	4/19/17	16w	2		NA
HCB genotype											
	Rx 98A	mut/mut	wt/wt	+/+	M	1/10/17	5/1/17	15w 6d	2		NA

fl-wt	Rx 98B	mut/mut	**mut/mut**	-/-	M	1/10/17	5/1/17	15w 6d	2	orai pres	NA
fl/fl	Rx 98C	mut/mut	wt/wt	+/+	M	1/10/17	5/1/17	15w 6d	2		
~fl/wt	Rx 98D	mut/mut	**mut/mut**	-/-	M	1/10/17	5/3/17	16w 1d	2	orai pres	NA
wt	Rx 98E	mut/mut	wt/wt	+/+	M	1/10/17	5/3/17	16w 1d	2		NA
fl/fl	Rx 99A	mut/mut	**wt/mut**	-/-	F	1/10/17	5/4/17	16w 2d	2	orai pres	
fl-wt	Rx 99B	mut/mut	wt/wt	+/+	F	1/10/17	5/4/17	16w 2d	2	no orai	NA

C. Derived cell lines, MSC first and then differentiated OB (not used in work shown)

MSC	Mouse ID	flox	cre	orai	sex	birth date	sac date	age at sac	genotype	WVU
	Rx 94A	mut/mut	**wt/mut**	-/-	F	12/10/16	4/10/17	17w 2d	(s-cre?)	
	Rx 95A	mut/mut	**mut/mut**	-/-	M	12/11/16	4/11/17	17w 2d	m-flox-cre	
	Rx 95B	mut/mut	**wt/mut**	-/-	M	12/11/16	4/11/17	17w 2d	m-flox-wt	
	Rx 95C	mut/mut	**wt/mut**	-/-	M	12/11/16	4/11/17	17w 2d	s-flox-?	
	Rx 95D	mut/mut	**wt/mut**	-/-	M	12/11/16	4/12/17	17w 1d	s-flox-?	
	Rx 97C	mut/mut	wt/wt	+/+	M	12/28/16	4/19/17	16w		
	Rx 98C	mut/mut	wt/wt	+/+	M	1/10/17	5/1/17	15w 6d		
	Rx 99A	mut/mut	**wt/mut**	-/-	F	1/10/17	5/4/17	16w 2d	orai pres	
	Rx 72A	**mut/mut**	**wt/mut**	-/-	F	7/20/16	1/25/17	27w		flox-
	Rx 72D	**mut/mut**	**wt/mut**	-/-	F	7/20/16	1/25/17	27w		flox-
	Rx 83E	mut/mut	wt/wt	+/+	F	9/12/16	3/21/17	27w 1d		flox-
	Rx 87B	mut/mut	wt/wt	+/+	F	9/14/16	3/21/17	26w 6d		

Ob	Mouse ID	flox	cre	orai	sex	birth date	sac date	age at sac	genotype	WVU
	Rx 90AKO	mut/mut	**wt/mut**	-/-	M	10/25/16	2/15/17	16w 1d		flox-
	Rx 90AZ	mut/mut	wt/wt	+/+	M	11/18/16	3/13/17	16w 3d		flox-
	Rx 90B	mut/mut	wt/wt	+/+	M	11/18/16	3/21/17	17w 4d		flox-
	Rx 95A	mut/mut	**mut/mut**	-/-	M	12/11/16	4/11/17	17w 2d	m-flox-cre	
	Rx 95C	mut/mut	**wt/mut**	-/-	M	12/11/16	4/11/17	17w 2d	s-?flox-cre	

Rx 98B	mut/mut	**mut/mut**	-/-	M	1/10/17	5/1/17	15w 6d orai pres	
Rx 98E	mut/mut	wt/wt	+/+	M	1/10/17	5/3/17	16w 1d	
Rx 99A	mut/mut	**wt/mut**	-/-	F	1/10/17	5/4/17	16w 2d orai pres	
Rx 72D	**mut/mut**	**wt/mut**	-/-	F	7/20/16	1/25/17	27w	flox-
Rx 87B	mut/mut	wt/wt	+/+	F	9/14/16	3/21/17	26w 6d	
Rx 87C	**mut/mut**	**wt/mut**	-/-	F	9/14/16	3/29/17	28w	flox-

2) Original data plotted in Fig 2 quantification of micro CT images.

Cortical Thickness Ribs

Group	ID	Measurements	AVE	SD
**Gr1**	90B (M) (17w)	154		
		154		
		154	154	
		148		
		166		
		166	160	
	97E (M) (16w)	189		
		190		
		172	184	
		178		
		166		
		172	172	
	99B (F) (16w)	166		
		154		
		160	160	
		148		
		148		
		160	152	
**Gr2**	95D (M) (17w)	178		
		190		
		148	172	
		148		
		172		
		178	166	

Cortical Density Ribs

Group	ID	Measurements	Ribs	AVE	SD
**Gr1**	90B (M) (17w)	94.3	A		
		90.38	A		
		95.9	A	93.53	
		93.93	B		
		94.5	B		
		96.01	B	94.81	
	97E (M) (16w)	88.1	C		
		89.71	C		
		92.39	C	90.07	
		94.22	D		
		90.2	D		
		90.88	D	91.77	
	99B (F) (16w)	84.77	E		
		88.99	E		
		88.79	E	87.52	
		84.12	F		
		86.52	F		
		85.54	F	85.39	
**Gr2**	95D (M) (17w)	73.5	A		
		71.94	A		
		73.95	A	73.13	
		74	B		
		72.53	B		
		73.65	B	73.39	

Cortical Density Vertebra

Gr1		
**1**	L5a	131
**2**	L5b	127
**3**	L5a	141
**4**	L5b	135
**5**	L5a	134
**6**	L5b	127

98D (M) (16w)	160	
	166	
	160	162
	178	
	178	
	172	176
99A (F) (16w)	184	
	172	
	172	176
	148	
	148	
	142	146

98D (M) (16w)	84.13	C	
	80.4	C	
	78.59	C	81.04
	87.34	D	
	88.15	D	
	88.42	D	87.97
99A (F) (16w)	87.26	E	
	89.15	E	
	89.4	E	88.60
	90.22	F	
	90.89	F	
	90.68	F	90.60

3) Original uncropped and unprocessed western blots from Fig 3. As per Plos instructions

Figure 4D top panel Western Blot

Method used: Proteins were separated on a 4-12% gradient bis-tris gel and transferred to polyvinylidene difluoride (PVDF) membranes. Target protein was detected by enhanced chemiluminescence on autoradiography film. Primary antibody rabbit anti-Orai1 antibody Alomone Labs (ACC-062, Jerusalem, Israel) (1:200). Secondary antibody horseradish peroxidase conjugated anti-rabbit (1:40,000, Jackson ImmunoResearch)

The position of the standards (colored, not labeled by antibody) is traced from the original pdf membrane Selected standards shown were identified by color.

Loading order and sample identity are labeled on the blot image

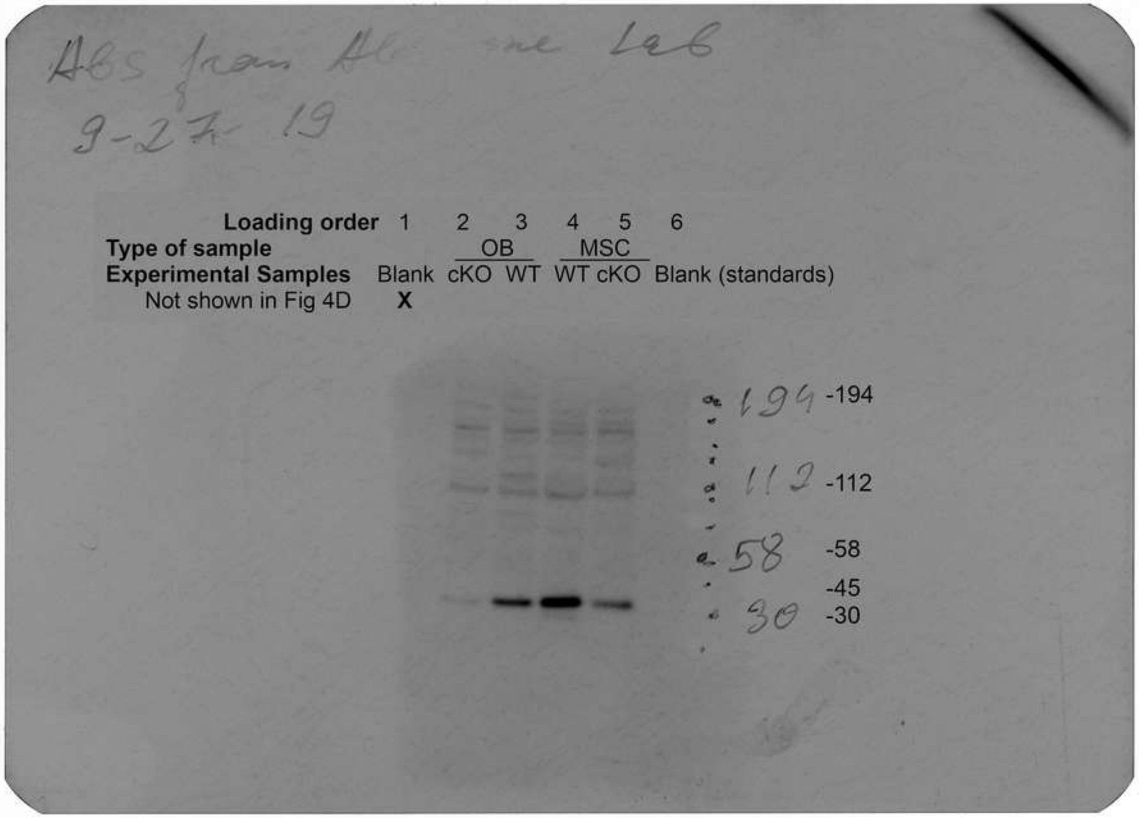

Actin Blot per Plos instructions

Figure 4D bottom panel Western blot

Method used: Proteins were separated on a 4-12% gradient bis-tris gel and transferred to polyvinylidene difluoride (PVDF) membranes. Target protein was detected by enhanced chemiluminescence on autoradiography film.
Mouse monoclonal beta-actin antibody A2228 (1:1,000, Sigma), with secondary horse radish peroxidase conjugated anti-mouse (1:40,000) was used. The actin re-blot was done three days later after removing the antibody with Restore Western Blot stripping solution (Fischer)

The position of the standards (colored, not labeled by antibody) is traced from the original pdf membrane
Selected standards shown were identified by color

Loading order and sample identity are labeled on the blot image

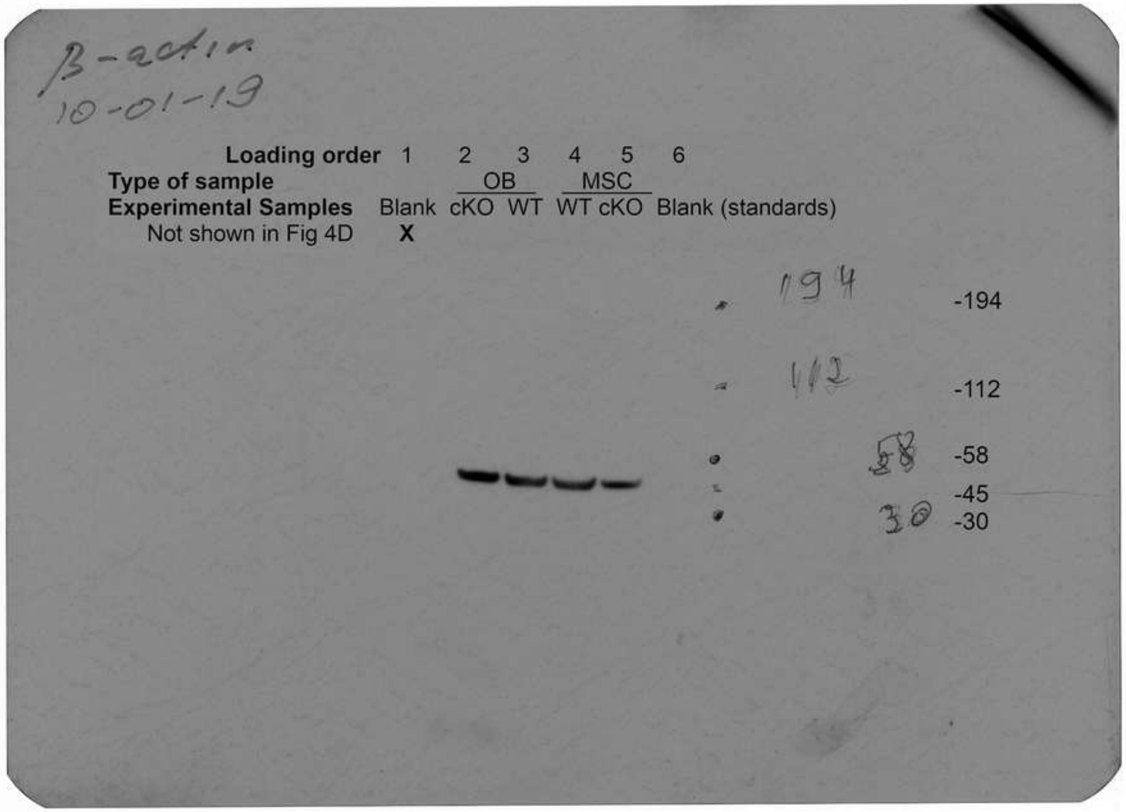

4) Original data for all micro CT measurements shown in Fig 3.

Bone Volume/Total Volume (BV/TV)

Wild Type	Orai1 cKO		
25.99	23.86		
25.61	13.98		
25.53	22.96		
23.80	13.49		
25.37	19.98		
23.69	19.53		
21.44	21.71		
ave			
sd			
24.50	19.36		n=7
1.62	4.13		
		p=	0.0099
		graphpad	

Trabecular Thickness (µm; Tb.Th)

Wild Type	Orai1 cKO		
50.26	49.79		
49.80	43.19		
50.16	50.00		
49.00	42.44		
51.16	46.62		
49.23	46.05		
50.29	46.49		
ave			
sd			n=7
49.98	46.37	p=	0.0077
0.72	2.90	graphpad	

Trabecular Number (mm-1; Tb.N)

Wild Type	Orai1 cKO		
0.0052	0.0048		
0.0051	0.0032		
0.0051	0.0046		
0.0049	0.0032		
0.0050	0.0043		
0.0048	0.0042		
0.0043	0.0047		
ave			
sd			n=7
0.0049	0.0041	p=	0.0189
0.0003	0.0007	graphpa	**

Trabecular spacing (Tb.Sp)

	Wild Type	Orai1 cKO	
	143.17	158.97	
	144.75	265.45	
	146.31	167.87	
	156.76	272.02	
	150.46	186.48	
	158.67	189.80	
	184.45	167.65	
ave	154.94	201.18	
sd	14.29	47.45	
		p=	0.0295
		graphpad	n=7

Fraction Surface Labeled

Wild Type	Orai1 cKO		
0.39	0.30		
0.34	0.30		
0.30	0.32		
0.35	0.41		
ave			
sd			
0.35	0.33		n=4
0.04	0.05		
		p=	NS
		graphpad	

Bone Formation (mm3/d; BFR)

Wild Type	Orai1 cKO		
10.29	5.93		
8.96	5.89		
7.82	6.29		
9.25	8.01		
ave			
sd			
9.08	6.53		n=4
1.02	1.00		
		p=	0.0118
		graphpad	

5) Original data for PCR measurements in Fig 5, beginning with list of all primers used in [12] and [16] and this manuscript

Table 1. PCR primers (Mouse)

ALP NM_007431.2 Product size 131 bp (Alkaline phosphatase)
F - 5'-ATCGGAACAACCTGACTGACCCTT-3' R - 5'-ACCCTCATGATGTCCGTGGTCAAT-3'
Col1a1 NM_007742.3 Product size 159 bp
F - 5'-TTCTCCTGGCAAAGACGGACTCAA-3' R - 5'-AGGAAGCTGAAGTCATAACCGCCA-3'
Osx NM_130458.3 Product size 146 bp (Sp7, Osterix**)**
F - 5'-GATGGCGTCCTCTCTGCTT-3' R - 5'-CGTATGGCTTCTTTGTGCCT-3'
RANKL NM_011613.3 Product size 83 bp
F - 5'-GCTCCGAGCTGGTGAAGAAA-3' R - 5'-CCCCAAAGTACGTCGCATCT-3'
LysM NM_001145920.1 Product size 105 bp
F - 5'-ATGATGACACTGCCACCTCTGAC-3' R - 5'-ACTGCCTGGGGTCTGAAAAAGG-3'
Atp1a2 (old name ATPa3) NM_178405.3 Osteoclast V ATPase Product size 163 bp
F - 5'-TGACCACAAGCTGTCCTTGGATGA-3' R - 5'-AAGCTGACGACAGAACTTGACCCA-3'
ATP6v0d2 NM_175406 Alternate osteoclast V-ATPase probe Product size 192bp
F - 5'-GGAAGCTGTCAACATTGCAGA -3' R - 5' -TCACCGTGATCCTTGCAGAAT-3'
Cathepsin-K NM_007802.3 Product size 174 bp
F - 5'-CAGCAGAGGTGTGTACTATG-3' R - 5'-GCGTTGTTCTTATTCCGAGC-3'
TRAP NM_001102405.1 Product size 174 bp
F - 5'-CACGAGAGTCCTGCTTGTC-3' R - 5'-AGTTGGTGTGGGCATACTTC-3'
PPARBЄI I ∈NM_011146.3 Product size 100 bp
F - 5'-CGCTGATGCACTGCCTATGA-3' R - 5'-AGAGGTCCACAGAGCTGATTCC-3'
Osteocalcin NM_007541.2, NM_001037939.1 Product Size: 118 bp (Bone Gla Protein, BGLAP)
F - 5`-ACCATCTTTCTGCTCACTCTGCTG-3` R - 5`-TATTGCCCTCCTGCTTGGACATGA-3`
Actin NM_007393.3 Product size 275 bp
F - 5'-GATATCGCTGCGCTGGTCGTC-3' R - 5'-ACGCAGCTCATTGTAGAAGGTGTG-3'
GAPDH NM_008084.3 Product size 184 bp (Glyceraldehyde-3-phosphate dehydrogenase)
F - 5'-GTTGTCTCCTGCGACTTCA -3' R - 5'-GGTGGTCCAGGGTTTCTTA-3'
Orai1 NM_175423.3 Product 192 bp
F - 5'- TACTTAAGCCGCGCCAAGCT-3' R - 5'GCAGGTGCTGATCATGAGGGC-3'
OPG NM_008764.3 Product 211 bp
F - 5'-TTTGCCTGGGACCAAAGTGAATGC-3' R - 5'-AAGAAGCTGCTCTGTGGTGAGGTT-3'
ATF4 (CREB2) product size 134 bp
F - 5'-CCTGAACAGCGAAGTGTTGG-3' R - 5'-TGGAGAACCCATGAGGTTTCAA-3'
RunX2 Product size 105
F - 5'-ATGATGACACTGCCACCTCTGAC-3' R - 5'-ACTGCCTGGGGTCTGAAAAAGG-3'

0.0119209207

MSC data

	GAPDH		Orai1		OCN		ALP		RUNX2		Col1 A1	
cKO												
95A-mut/mut,M	17.54	0.687770909	29.72	0.000215504								
95A-mut/mut,M	17.58	0.668963777	29.82	0.000206725								
95D-wt/mut,M	18.33	0.397768242	28.03	0.001202289								
95D-wt/mut,M	18.3	0.406126198	27.77	0.001410087	26.72	0.001153313						
72A-wt/mut,F	19.42	0.186856156	29.41	0.000983355	26.79	0.001145347					22.42	0.132127255
72A-wt/mut,F	19.4	0.189464571	29.6	0.000850147	27.83	0.00085606					22.43	0.139660892
72D-wt/mut,F	19.62	0.162667732	28.69	0.001860621	27.86	0.000804288					27.68	0.003799433
72D-wt/mut,F	19.61	0.163799175	28.72	0.001809742	22.61	0.018971795					27.48	0.003594483
95A-CRE	16.96	1.028113827	26.72	0.001153313	22.58	0.01991501	23.89	0.003825861	23.91	0.008088007	20.65	0.077482
95A-CRE	17.02	0.986232704	26.79	0.001145347			24.13	0.003670011	24.03	0.008200912	20.83	0.075363
95C-CRE	17.64	0.641712949	27.83	0.00085606			28.84	0.000372622	21.66	0.053289681	19.84	0.188156
95C-CRE	17.58	0.668963777	27.86	0.000804288			28.74	0.000410594	21.68	0.052556026	19.88	0.183011
95D-CRE	16.89	1.079228237	22.61	0.018971795			22.4	0.02005353	21.07	0.041810236	17.32	0.562529
95D-CRE	16.93	1.049716684	22.58	0.01991501		0.007140969	22.5	0.01991501	21.19	0.039554894	17.31	0.582367
	Av	0.88517087		0.003670306		0.009535174		0.008041271		0.033916626		0.194808938
n=14	sd	0.19899326		0.006701963				0.009372225		0.020713557		0.209139422
WT				p=0.018		p=0.0379		p=0.0012		p=0.94 ns		p= 0.07 (ns)
97C	16.32	1.602139755	22.8	0.011202775								note two outlie
	16.24	1.693490625	22.77	0.010821168								
83E	16.77	1.172834949	22.46	0.019370433	21.24	0.03794359						
	16.72	1.214194884	22.44	0.018971795	21.18	0.040666933						
83E-WT	16.52	1.394743666	21.24	0.03794359	19.44	0.211686328	17.58	0.133971683	22.08	0.019103754	17.85	0.358489
83E-WT	16.56	1.356604327	21.18	0.040666933	19.41	0.207329886	17.53	0.142595464	21.93	0.020760716	17.83	0.356013
98B-WT	17.2	0.870550563	19.44	0.211686328	18.1	0.757858283	20.66	0.095391201	21.6	0.044501569	18.28	0.444421
98B-WT	17.14	0.907519155	19.41	0.207329886	18.06	0.768437591	20.7	0.090245575	21.48	0.047038961	18.37	0.406126
97C-WT	17.7	0.615572207	18.1	0.757858283			20.57	0.038207509	21.78	0.039281668	18.79	0.312083
97C-WT	17.68	0.624165274	18.06	0.768437591			20.6	0.038207509	21.84	0.036651092	18.63	0.339151
	Av	0.874882305		0.208428878				0.089769823		0.034556293		0.369380436
n=10	sd	0.343060924		0.302266912				0.044929998		0.011920921		0.047907344
						0.337320435						

Osterix PCR Data

Replic	Dye	Threshold	Primers	cDNA	Ct (dR)	Proportion
1	SYBR	51.519	GAPDH	97C-WT	16.57	
2	SYBR	51.519	GAPDH	97C-WT	16.65	
3	SYBR	51.519	GAPDH	83E-WT	15.36	
4	SYBR	51.519	GAPDH	83E-WT	15.52	
5	SYBR	51.519	GAPDH	95D-KO	16.91	
6	SYBR	51.519	GAPDH	95D-KO	17.12	
7	SYBR	51.519	GAPDH	95C-KO	18.25	
8	SYBR	51.519	GAPDH	95C-KO	18.52	
9	SYBR	51.519	Osterix	97C-WT	20.8	0.0533
10	SYBR	51.519	Osterix	97C-WT	20.76	0.0579
11	SYBR	51.519	Osterix	83E-WT	18.76	0.0947
12	SYBR	51.519	Osterix	83E-WT	18.63	0.1158
13	SYBR	51.519	Osterix	95D-KO	21.99	0.0296
14	SYBR	51.519	Osterix	95D-KO	22.07	0.0324
15	SYBR	51.519	Osterix	95C-KO	25.04	0.0090
16	SYBR	51.519	Osterix	95C-KO	25.01	0.0111
17	SYBR	51.519	ATF-4	97C-WT	18.73	0.2238
18	SYBR	51.519	ATF-4	97C-WT	18.83	0.2207
19	SYBR	51.519	ATF-4	83E-WT	18.01	0.1593
20	SYBR	51.519	ATF-4	83E-WT	17.95	0.1856
21	SYBR	51.519	ATF-4	95D-KO	19.81	0.1340
22	SYBR	51.519	ATF-4	95D-KO	20.03	0.1330
23	SYBR	51.519	ATF-4	95C-KO	20.93	0.1560
24	SYBR	51.519	ATF-4	95C-KO	20.98	0.1817
25	SYBR	51.519	ATF-4-1	97C-WT	18.28	0.3057
26	SYBR	51.519	ATF-4-1	97C-WT	18.45	0.2872
27	SYBR	51.519	ATF-4-1	83E-WT	17.4	0.2432
28	SYBR	51.519	ATF-4-1	83E-WT	17.35	0.2813
29	SYBR	51.519	ATF-4-1	95D-KO	19.73	0.1416
30	SYBR	51.519	ATF-4-1	95D-KO	19.32	0.2176
31	SYBR	51.519	ATF-4-1	95C-KO	20.23	0.2535
32	SYBR	51.519	ATF-4-1	95C-KO	20.36	0.2793
33	SYBR	51.519	H2O	H2O	No Ct	
34	SYBR	51.519	H2O	H2O	No Ct	

WT	KO
0.0533	0.0296
0.0579	0.0324
0.0947	0.009
0.1158	0.0111

ATF PCR data

WT	KO
0.2238	0.134
0.2207	0.133
0.1593	0.156
0.1856	0.1817

6) Quantitative data for Fig 6

Stain		wt	ko	wt	ko		Von Kossa	Mean	SD	n=4
VK		note -- all are inverses		direct			WT	126.00	18.46	p = 0.002
	1	146	207	110.00	49.00		KO	47.75	2.22	
	2	145	206	111.00	50.00					
	3	109	209	147.00	47.00					
	4	120	211	136.00	45.00					
mean				126.00	47.75					
sd				18.46	2.22					

							Alk Phos	Mean	SD	n=4
Alk phos										
	1	109	183	147.00	73.00		WT	134.00	12.30	p<0.0001
	2	114	186	142.00	70.00		KO	73.25	2.36	
	3	133	181	123.00	75.00					
	4	132	181	124.00	75.00					
mean				134.00	73.25					
sd				12.30	2.36					

							ORO	Mean	SD	n=4
ORO										
	1	185	183	71.00	73.00		WT	61.75	11.30	NS
	2	185	183	71.00	73.00		KO	62.75	12.01	
	3	199	206	57.00	50.00					
	4	208	201	48.00	55.00					
mean				61.75	62.75					
sd				11.30	12.01					
